# F11R Is a Novel Monocyte Prognostic Biomarker for Malignant Glioma 

**DOI:** 10.1371/journal.pone.0077571

**Published:** 2013-10-11

**Authors:** Winnie W. Pong, Jason Walker, Todd Wylie, Vincent Magrini, Jingqin Luo, Ryan J. Emnett, Jaebok Choi, Matthew L. Cooper, Malachi Griffith, Obi L. Griffith, Joshua B. Rubin, Gregory N. Fuller, David Piwnica-Worms, Xi Feng, Dolores Hambardzumyan, John F. DiPersio, Elaine R. Mardis, David H. Gutmann

**Affiliations:** 1 Department of Neurology, Washington University School of Medicine, St. Louis, Missouri, United States of America; 2 The Genome Institute, Washington University School of Medicine, St. Louis, Missouri, United States of America; 3 Division of Biostatistics, Washington University School of Medicine, St. Louis, Missouri, United States of America; 4 Division of Oncology, Department of Medicine, Washington University School of Medicine, St. Louis, Missouri, United States of America; 5 Department of Pediatrics, Division of Pediatric Hematology/Oncology, Washington University School of Medicine, St. Louis, Missouri, United States of America; 6 Department of Pathology, MD Anderson Cancer Center, Houston, Texas, United States of America; 7 BRIGHT Institute and Mallinckrodt Institute of Radiology, Washington University School of Medicine, St. Louis, Missouri, United States of America; 8 Department of Stem Cell Biology and Regeneration, Cleveland Clinic Foundation, Cleveland, Ohio, United States of America; University Hospital of Heidelberg, Germany

## Abstract

**Objective:**

Brain tumors (gliomas) contain large populations of infiltrating macrophages and recruited microglia, which in experimental murine glioma models promote tumor formation and progression. Among the barriers to understanding the contributions of these stromal elements to high-grade glioma (glioblastoma; GBM) biology is the relative paucity of tools to characterize infiltrating macrophages and resident microglia. In this study, we leveraged multiple RNA analysis platforms to identify new monocyte markers relevant to GBM patient outcome.

**Methods:**

High-confidence lists of mouse resident microglia- and bone marrow-derived macrophage-specific transcripts were generated using converging RNA-seq and microarray technologies and validated using qRT-PCR and flow cytometry. Expression of select cell surface markers was analyzed in brain-infiltrating macrophages and resident microglia in an induced GBM mouse model, while allogeneic bone marrow transplantation was performed to trace the origins of infiltrating and resident macrophages. Glioma tissue microarrays were examined by immunohistochemistry, and the Gene Expression Omnibus (GEO) database was queried to determine the prognostic value of identified microglia biomarkers in human GBM.

**Results:**

We generated a unique catalog of differentially-expressed bone marrow-derived monocyte and resident microglia transcripts, and demonstrated that brain-infiltrating macrophages acquire F11R expression in GBM and following bone-marrow transplantation. Moreover, mononuclear cell F11R expression positively correlates with human high-grade glioma and additionally serves as a biomarker for GBM patient survival, regardless of GBM molecular subtype.

**Significance:**

These studies establish F11R as a novel monocyte prognostic marker for GBM critical for defining a subpopulation of stromal cells for future potential therapeutic intervention.

## Introduction

Survival for adults with the malignant brain tumor, glioblastoma multiforme (GBM), remains poor despite decades of advancements in surgery, radiation, and chemotherapy. One underexplored strategy for treating these cancers is the targeting of stromal cell types in the tumor microenvironment. In this regard, microglia and macrophages may serve as tractable targets for stroma-directed therapy, as they comprise 30-50% of the cells in both benign and malignant gliomas [1,2]. In previous genomic studies, glioma outcome and progression were shown to correlate with macrophage and microglia gene expression [3], while polymorphisms in the microglial *CX3CR1* chemokine receptor locus were associated with improved patient survival [4]. Furthermore, pharmacologic or genetic inhibition of microglial function reduces tumor growth in experimental rodent glioma models [2,5–9]. 

One of the barriers to developing glioma stromal therapies is the paucity of suitable reagents to characterize the spectrum of macrophage populations in health and disease. Although recent reports have established that the tissue origins for mouse brain (resident) microglia and bone-marrow derived monocytes (BMDM) are distinct [10], mouse and human brain tumors harbor potentially distinct and functionally important subpopulations of infiltrating monocytes and resident microglia. To identify new macrophage markers relevant to high-grade glioma, we sought to discover BMDM- and brain microglia-specific transcripts to enable an analysis of the role of these mononuclear cell populations in GBM. 

In this study, we leveraged four converging analysis methods across two complementary platforms to identify a series of differentially-expressed BMDM and brain microglia transcripts. Following validation by real-time quantitative RT-PCR and flow cytometry, we selected two representative differentially-expressed BMDM and microglia surface markers (SELL and F11R) to demonstrate that infiltrating BMDM in induced murine malignant glioma acquire F11R expression, which was verified using allogeneic bone marrow transplantation. To establish the clinical relevance of F11R to human GBM, we show that F11R expression correlates positively with glioma malignancy grade as well as correlates negatively with patient survival independent of GBM molecular subtype. 

## Materials and Methods

### Ethics Statement

All mice were maintained in strict accordance with recommendations in the Guide for the Care and Use of Laboratory Animals of the National Institutes of Health and active animal studies protocols approved by the Animal Studies Committee at the Washington University School of Medicine (Protocol Numbers: 20110111 and 20120058) and the Institutional Animal Care And Use Committee (Protocol Number: 2010-0268) at the Cleveland Clinic Foundation. All surgeries were performed under Ketamine (100mg/kg) and Xylazine (10mg/kg) anesthesia, and all efforts were made to minimize suffering. Animals were also provided 0.25% Marcaine (Bupivacaine) in the volume of approximately 0.1mL/25g post-surgery to provide pain relief. 

All human samples were collected on a protocol approved by the Institutional Review Board at the Washington University School of Medicine (Permit Number: 201103323) to comply with ethical standards as well as government and institutional regulations. Tissue microarrays cores were received by the Tissue Procurement Core Facility and Tumor Bank as de-identified specimens, and consent was waived.

### Experimental mouse models

*Ntv-a*;*Ink4a-Arf-/-*;Gli-luc mice that develop high-grade gliomas following intracranial RCAS-PDGFB injection at 6 weeks of age were imaged by luciferase bioluminescence (BLI) prior to tissue collection at 3 months of age [11]. Control naïve mice were age and gender matched and not injected with RCAS. Whole control brains (from naïve mice not receiving RCAS injections) and tumor masses from glioma-bearing mice were isolated and collected for flow cytometry analyses.

Allogeneic bone marrow transplantation (BMT) was employed to induce graft-versus-host-disease (GVHD)[12]. Briefly, bone marrow from B6.SJL-*Ptprc* ^*a*^ *Pepc^b^/BoyJ* mice (Jackson Laboratory, CD45.1, H-2K^b^) were T cell depleted (TCD) using CD90.2 microbeads and an AutoMACS (Miltenyi Biotec GmbH, Auburn, CA), and injected intravenously into BALB/c recipient mice (Jackson Laboratory, CD45.2, H-2K^d^) preconditioned with 925 cGy total body irradiation (TBI) prior to BMT. Following BMT, delayed lymphocyte infusions (DLI) from C57BL/6 (CD45.2, H-2K^b^) were injected on day 11 post-BMT. For DLI, mouse T cells (total CD4+ and CD8+ T cells) were isolated from mouse spleens using Miltenyi microbeads and an AutoMACS (Miltenyi Biotech, Auburn, CA). Control mice received BMT only, without DLI. Tissues were collected for flow cytometry analysis at 1, 2, and 3 weeks post-DLI (n=6 per treatment per time point). All experiments were performed at least twice on independently-generated mouse cohorts.

#### Flow cytometry and fluorescence-activated cell sorting (FACS)

Brain and bone marrow were collected from anesthetized and Ringer’s solution-perfused mice, and mononuclear cells were isolated for antibody-mediated flow cytometry and FACS (Table **S1**) [13]. For intracellular staining, surface-stained cells were fixed and permeabilized with the BD Cytofix/Cytoperm Fixation/Permeabilization Kit (BD Biosciences). Nonspecific staining and gating was determined using isotype and fluorescence minus one (FMO) controls. Forward Scatter (FSC) and Side Scatter (SSC) were used to determine viable cells, and appropriate controls were employed for compensation and gating [14]. BMDM were gated on cells that were CD11b^+^ CD115^+^ Ly6G^-^, while brainstem microglia (BSM) were CD11b^+^ CD45^low^ Ly6G^--^ to generate near pure (>99.9%) populations of BMDM and BSM cells. FACS samples were placed into TRIzol (Life Technologies Corporation, Carlsbad, CA) for RNA extraction without any intervening *in vitro* tissue culture adaptation or exposure to growth factors. . 

#### RNA protocols

Total RNA was isolated from sorted cell pellets using TRIzol-chloroform extraction, resuspended in Ambion Nuclease-free water (Life Technologies), snap frozen, and stored at -80°C. RNA quality and yield were assayed using the Agilent Eukaryotic Total RNA 6000 and the BioRad Experion, then quantified using the Quant-iT™ RNA assay kit on a Qubit™ Fluorometer (Life Technologies). 

#### Sequencing and microarray platforms

The Ovation® RNA-Seq method was employed for cDNA synthesis, and 500ng cDNA were used for Illumina library construction with the Illumina paired-end LT indexing protocol [15,16]. Each library was sequenced on the Illumina HiSeq, generating between 15-22Mbp per lane of 100 basepair paired-end reads. For microarray analyses, cDNA prepared from total RNA (NuGEN Ovation WTA Pico V.2, NuGEN Technologies, Inc., San Carlos, CA) was used to probe the Mouse Exon 1.0ST array (Affymetrix, Santa Clara, CA).

#### RNA-seq and microarray analysis methods

Six mouse samples were sequenced from independently-generated biological replicates that included three samples of BSM and three samples of BMDM (six lanes of Illumina data sequenced in total). Corresponding RNA-Seq paired-end reads were processed using the TopHat suite [17] with Cufflinks [18,19] as well as ALternative EXpression Analysis by Sequencing (ALEXA-Seq) [20]. 

Microarray data were analyzed with Partek^®^ Discovery Suite software (version 6.6, Partek Inc., St. Louis, MO) and Aroma (http://www.aroma-project.org/). Additional details are provided in Methods **S1**.

A fold-change rank for every gene was generated based on each independent analysis as well as a mean fold-change rank across the four independent analyses (Cufflinks, ALEXA-Seq, Aroma, and Partek), culminating in a final list based on the mean fold-change rank and significance (<0.05 False Discovery Rate, FDR).

#### Quantitative real time polymerase chain reaction (qPCR).

cDNA was synthesized using the Omniscript reverse transcription kit (Qiagen, Alameda, CA). qPCR was performed using the Bio-Rad CFX96 Real-Time System (Bio-Rad Laboratories Inc., Hercules, CA) with SYBR Green detection (Life Technologies Corporation, Carlsbad, CA) or TaqMan probe-based chemistry with pre-designed TaqMan® Gene Expression Assays (Life Technologies). Primer sequences were designed with Primer-BLAST (NCBI, http://www.ncbi.nlm.nih.gov/tools/primer-blast/) to span exon-exon junctions and target known splice variants (Table **S2**). The ΔΔCT method was used to calculate fold expression changes. Results were analyzed with two-tailed Student’s T-test in Graphpad Prism 5 software and displayed as mean ± standard error of the mean (SEM).

#### Immunohistochemistry

Tissue microarrays containing cores from normal brain and tumor samples over a range of glioma malignancy grades were immunostained with appropriate antibodies (Table **S1**) prior to 3,3’-diaminobenzidine development. Images were acquired on a Nikon Eclipse E600 microscope (Nikon Corporation, Tokyo, Japan) equipped with a Leica EC3 optical camera and Leica Application Suite 2.10 (Leica Microsystems, Wetzlar, Germany). Investigators were blind to clinical grades, and percentages of only the positive mononuclear cells stained by immunohistochemistry were calculated using the total number of cells in each image (hematoxylin nuclear staining). The identities of the F11R+ cell types in the tumors were independently assessed using cell type-specific antibodies to verify that only mononuclear cells were included in the analysis (Figure **S1**). Data were analyzed with Graphpad Prism 5 software using ANOVA, and outliers were excluded using Grubbs’ test. 

#### Statistical analyses

Statistical analyses were performed using Graphpad Prism 5 statistical software. Group results are expressed as mean values ± SEM. Data between two groups were compared using unpaired two-tailed Student’s T-tests. Data among multiple groups were compared using Kruskal-Wallis test followed by Dunn’s Multiple Comparison testing, with a significance level set at p<0.05.

#### Survival outcome analyses

The Cancer Genome Atlas (TCGA) [21] subtype genes were matched in the NCBI Gene Expression Omnibus (GEO) [22] dataset accession GSE16011 [23] by Entrez ID, and after merging, each GBM sample (159 out of 276 total samples) was assigned to a TCGA subtype (Classical, Mesenchymal, Neural, or Proneural) based on the 10-nearest neighbor method. Each candidate gene expression level was associated with a survival outcome using the Cox proportional hazard model; the resulting hazard ratio (HR), its associated p-value, and a 95% confidence interval (CI) were reported. Candidate gene expression levels were dichotomized by median expression levels (across all samples). Kaplan-Meier curves were produced and log rank tests employed to compare survival differences between the low/high-expression groups. Multivariate Cox models based on gene expression, one continuous, and the other dichotomized by the median (High vs. Low), were each used to assess the prognostic ability of each candidate gene after accounting for TCGA molecular subtype.

## Results

To identify transcripts that potentially distinguish infiltrating monocytes from brain microglia, we isolated and examined gene expression of BMDM (CD11b^+^ CD45^high^ CD115^+^ Ly6G^-^ cells) and brainstem microglia (BSM) (CD11b^+^ CD45^low^ CD115^low^ Ly6G^-^ cells) pooled from 6-week-old C57BL/6 male mice (ten mice/set) by FACS. Despite low RNA yields (0.4-100ng of total RNA), overall good quality RNA was obtained (Agilent RNA Integrity Number, RIN=7.6-9.0) [24], and the resulting cDNA yields (4.9-5.8µg) were used to perform parallel Affymetrix Mouse Exon 1.0ST microarray and Illumina RNA-sequencing (Figure **S2**, Table **S3** and **S4**). 

**Figure 1:**
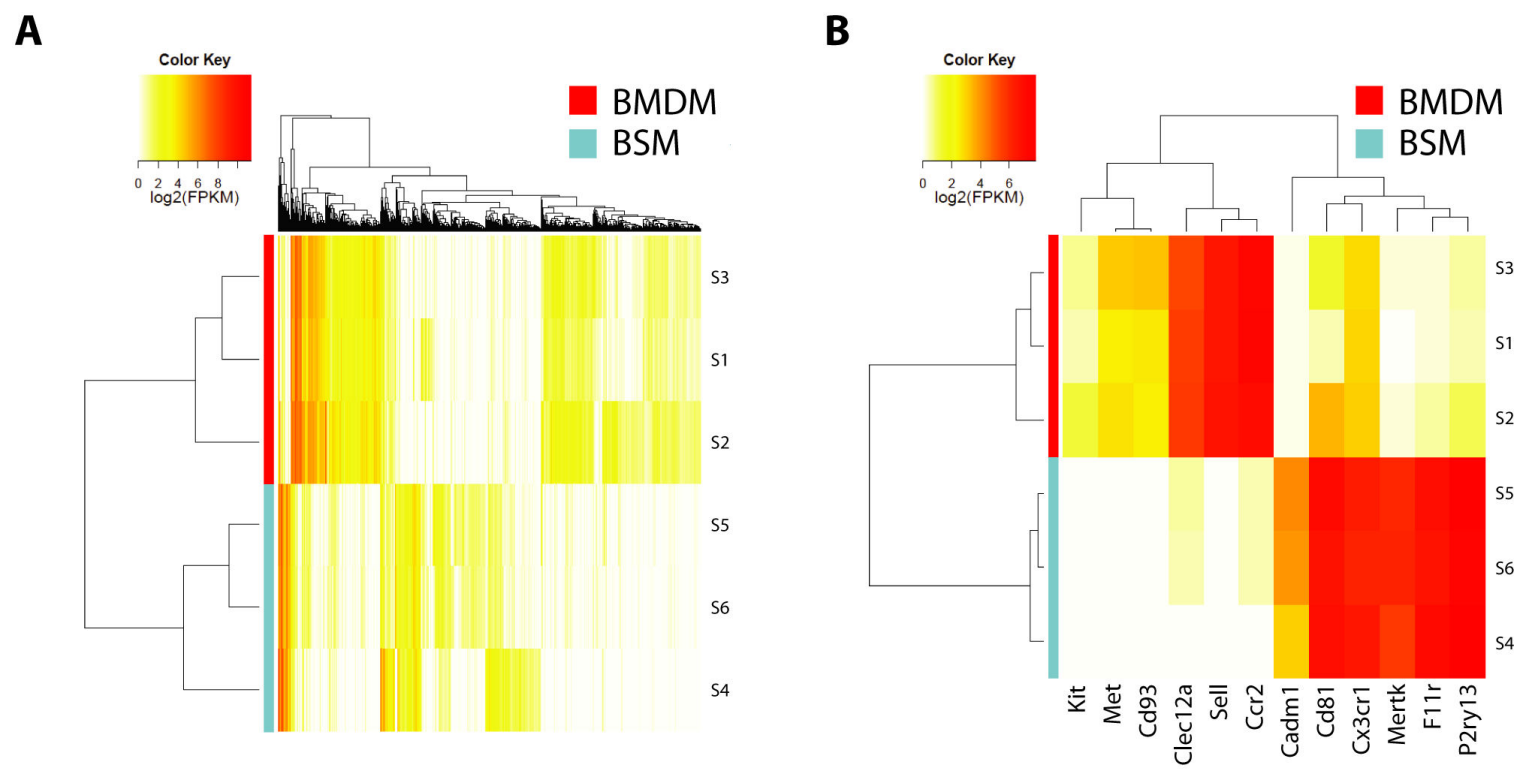
Differentially-expressed brainstem microglia (BSM) and bone marrow monocyte (BMDM) transcripts. (**A**) A heat map was generated from unsupervised hierarchical clustering analysis that included all genes expressed (FPKM >1 in ≥1/6 samples). (**B**) Candidate genes selected for further validation for comparison to CX3CR1 and CCR2.

Complementary analysis pipelines Cufflinks and ALEXA-Seq for RNA-Seq [18–20,25] and Aroma and Partek for microarray data [26–28] were employed to analyze each platform. Fold changes and q-values were calculated for each gene, and a mean fold-change rank was generated by merging the four independent analyses. High-confidence gene lists were subsequently generated that included only differentially-expressed genes identified to have q-values <0.05 by at least three of the four analysis methods (Table **S5**). A global unsupervised analysis, excluding genes not expressed or not variably expressed by Cufflinks analysis, demonstrated clear separation of BMDM from BSM samples (Figure **1A**). 

**Figure 2:**
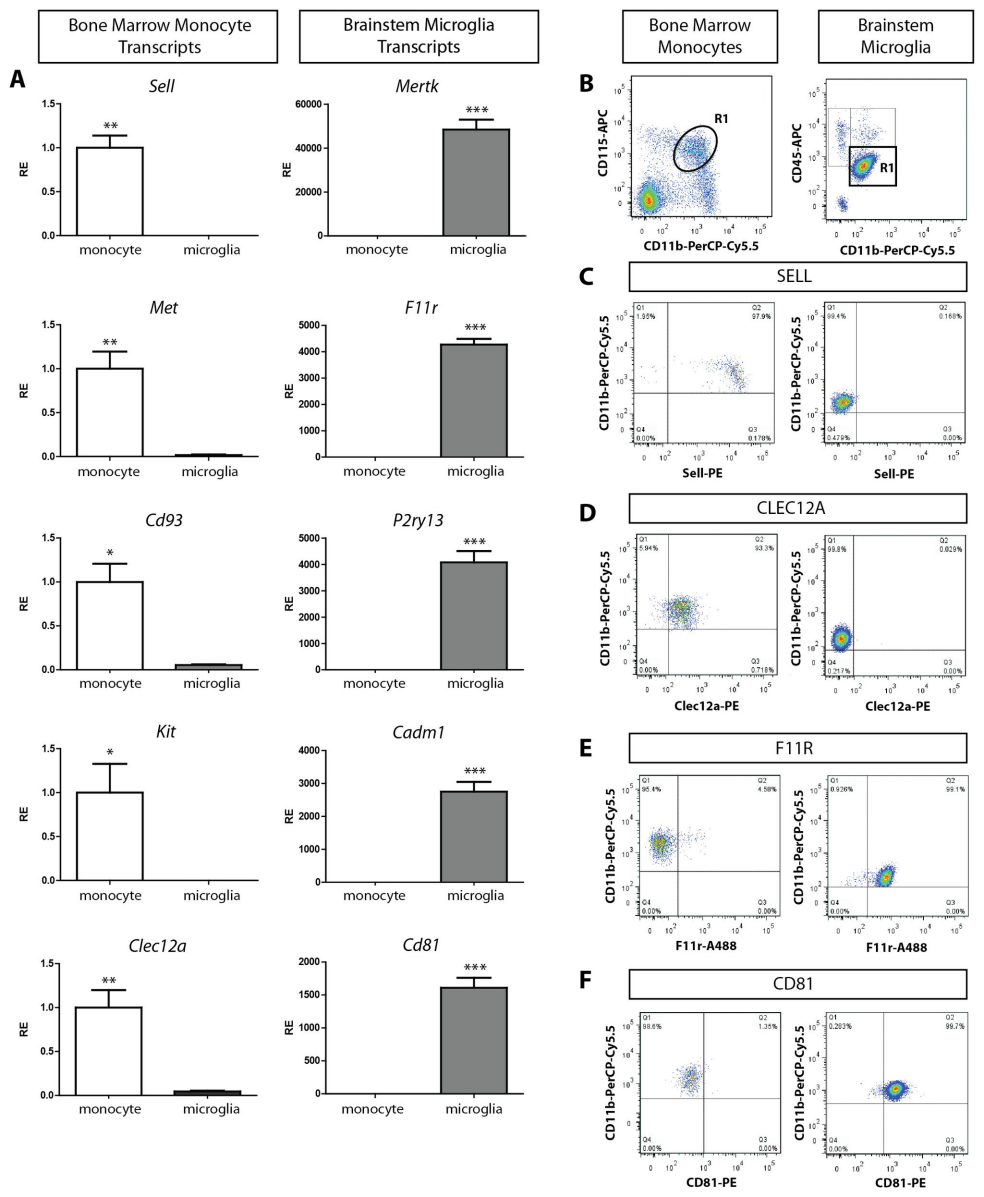
Transcript and protein validation of genes differentially expressed between BMDM and BSM. (**A**) BMDM-enriched transcripts, including *Sell* (p=0.0019), *Met* (p=0.0074), *Cd93* (p=0.0104), *Kit* (p=0.0377), and *Clec12a* (p=0.0049), were more highly expressed in independently-generated BMDM (n=5) relative to BSM (n=6) using TaqMan and SYBR Green qPCR. BSM-enriched transcripts, including *Mertk* (p=0.0001), *F11r* (p<0.0001), *P2ry13* (p=0.0002), *Cadm1* (p=0.0002), and *Cd81* (p=0.0001), were more highly expressed in BSM. (**B**) Flow cytometry analysis of BMDM (CD11b^+^ and CD115^+^ cells; R1, left panel) and BSM (CD11b^+^ CD45^low^ cells; R1, right panel) verified that SELL (**C**) and CLEC12A (**D**) were detected on BMDM, and not on BSM, while F11R (**E**) and CD81 (**F**) were detected on BSM and not BMDM. *, p<0.05; **, p<0.01; ***, p<0.001.

We next prioritized candidate transcripts based on their expression of protein products potentially amenable to flow cytometry analysis (cell surface markers), and selected ten transcripts of predicted membrane-associated proteins (5 BSM and 5 BMDM; Table **1**-**2**) for real-time quantitative PCR (qPCR) verification (Figure **1B** and Figure **2A**). We secondarily validated two representative transcripts for BSM (F11R and CD81) and BMDM (SELL and CLEC12A) by flow cytometry (Figure **2B-F**). Since CD81 resides inside the cell membrane of unstimulated microglia, and CLEC12A is expressed at relatively low levels in monocytes, we chose F11R and SELL as representative differentially-expressed flow cytometry markers to study monocyte infiltration in murine glioblastoma.

**Table 1:** Genes of predicted membrane-associated proteins of brain microglia across a wide range of fold changes are highlighted relative to *Cx3cr1*.

**Entrez ID**	**Gene Symbol**	**Full Name**	**Fold Change (Cuffdiff)**	**Q value (Cuffdiff)**	**Fold Change (Partek)**	**Q Value (Partek)**
17289	*Mertk*	c-mer proto-oncogene tyrosine kinase	1009.82	1.04E-13	264.86	2.93E-07
16456	*F11r*	F11 receptor	1226.64	1.55E-12	152.41	6.21E-06
74191	*P2ry13*	purinergic receptor P2Y, G-protein coupled 13	808.00	3.90E-12	185.89	2.26E-04
54725	*Cadm1*	cell adhesion molecule 1	271.93	1.81E-14	22.64	2.26E-04
12520	*Cd81*	CD81 antigen	98.16	6.95E-14	46.11	4.77E-04
13051	*Cx3cr1*	chemokine (C-X3-C) receptor 1	29.39	3.75E-05	7.54	4.84E-04

**Table 2:** Genes of predicted membrane-associated proteins of bone marrow monocytes across a wide range of fold changes are highlighted relative to *Ccr2*.

**Entrez ID**	**Gene Symbol**	**Full Name**	**Fold Change (Cuffdiff)**	**Q value (Cuffdiff)**	**Fold Change (Partek)**	**Q Value (Partek)**
20343	*Sell*	selectin, lymphocyte	3643.55	0.00E+00	284.09	4.65E-04
12772	*Ccr2*	chemokine (C-C motif) receptor 2	158.34	2.50E-10	178.05	3.91E-04
17295	*Met*	met proto-oncogene	257.26	1.22E-08	60.67	1.76E-03
17064	*Cd93*	CD93 antigen	83.62	2.84E-07	23.03	1.25E-02
232413	*Clec12a*	C-type lectin domain family 12, member a	38.85	3.26E-06	92.36	1.35E-05
16590	*Kit*	kit oncogene	42.41	2.84E-04	2.05	3.39E-02

**Figure 3:**
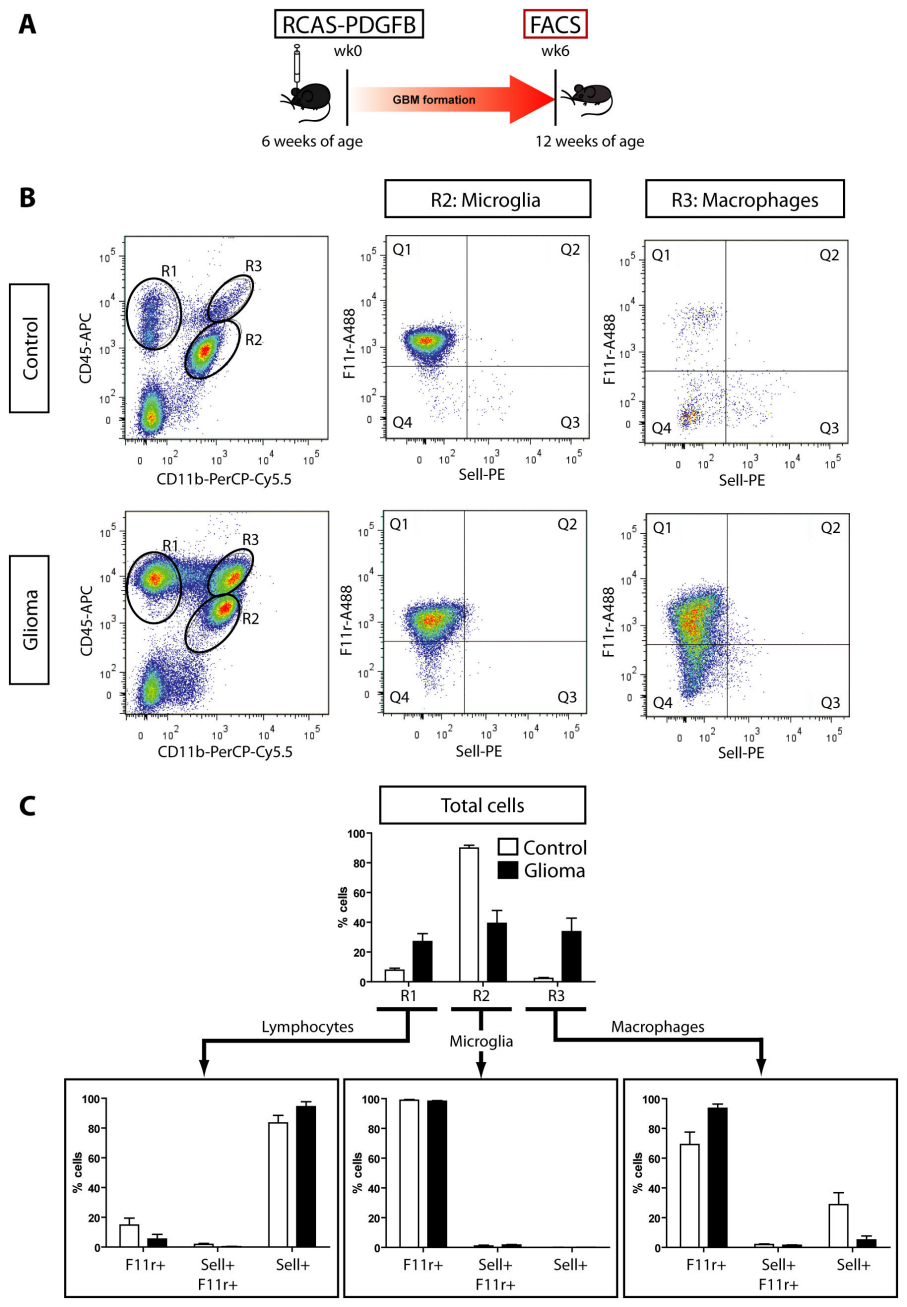
High-grade murine gliomas contain F11r^+^ microglia and macrophages. (**A**) Ntv-a Ink4a-Arf-/-;Gli-luc mice develop tumors following intracranial RCAS-PDGFB injection (n=4). Sell and F11r expression was examined by flow cytometry in lymphocytes (R1, CD11b^-^ CD45^+^ cells), microglia (R2, CD11b^+^ CD45^low^ cells), and macrophages (R3, CD11b^+^ CD45^high^ cells) within the tumor and in control naïve brains (n=4) in four separate experiments. (**B**) More F11r^+^ microglia and macrophages were identified in the gliomas relative to the control brains, most notably in the expansion of the R3 population (34% of positively labeled cells), which is typically a very small percentage in control brains (<2%). The majority of the labeled macrophages in the glioma are positive for F11r only (Q1; 94%), with few cells infiltrating the glioma positive for Sell only (Q3; 5%) or double positive for both F11r and Sell (Q2; 1.4%). (C) Bar graphs illustrate the mean (SEM) percentage and SEM for each immune cell population as well as their corresponding F11r and Sell surface expressions. White bars = control, black bars = glioma.

Since F11r and Sell are expressed in <2% of the total cells isolated from bone marrow and brain, respectively, we first sought to determine whether F11r and Sell would retain their distinctive expression between infiltrative BMDM and resident microglia populations in the setting of induced murine malignant glioma using *Ntv-a Ink4a-Arf-/-*;Gli-luc mice injected with RCAS-PDGFB (Figure **3A**) [11]. Whereas control naïve mice do not form gliomas nor contain significant numbers of CD11b^+^ CD45^high^ BMDM cells (R3; <2% cells) (Figure **S3**, Figure **3B**), murine gliomas exhibit increased numbers of lymphocytes (R1; 27% cells) as well as CD11b^+^ CD45^low^ cells (R2, microglia; 39% cells) and CD11b^+^ CD45^high^ cells (R3, macrophages; 34% cells). While CD11b^+^ CD45^low^ microglia were >99% F11r^+^, the population of macrophages was not exclusively Sell^+^. Surprisingly, the majority of these glioma-associated CD11b^+^ CD45^high^ macrophages were also F11r^+^ (94%, Q1) (Figure **3B**). Because fewer than 6% of these glioma-associated F11r^+^ cells were dendritic cells, B cells, or NK cells as determined by flow cytometry using CD3, CD4, CD11c, CD8a, CD19, CD103, and NK1.1 antibodies (data not shown), we hypothesized that Sell^+^ BMDM acquire F11r expression following brain infiltration in GBM. 

**Figure 4:**
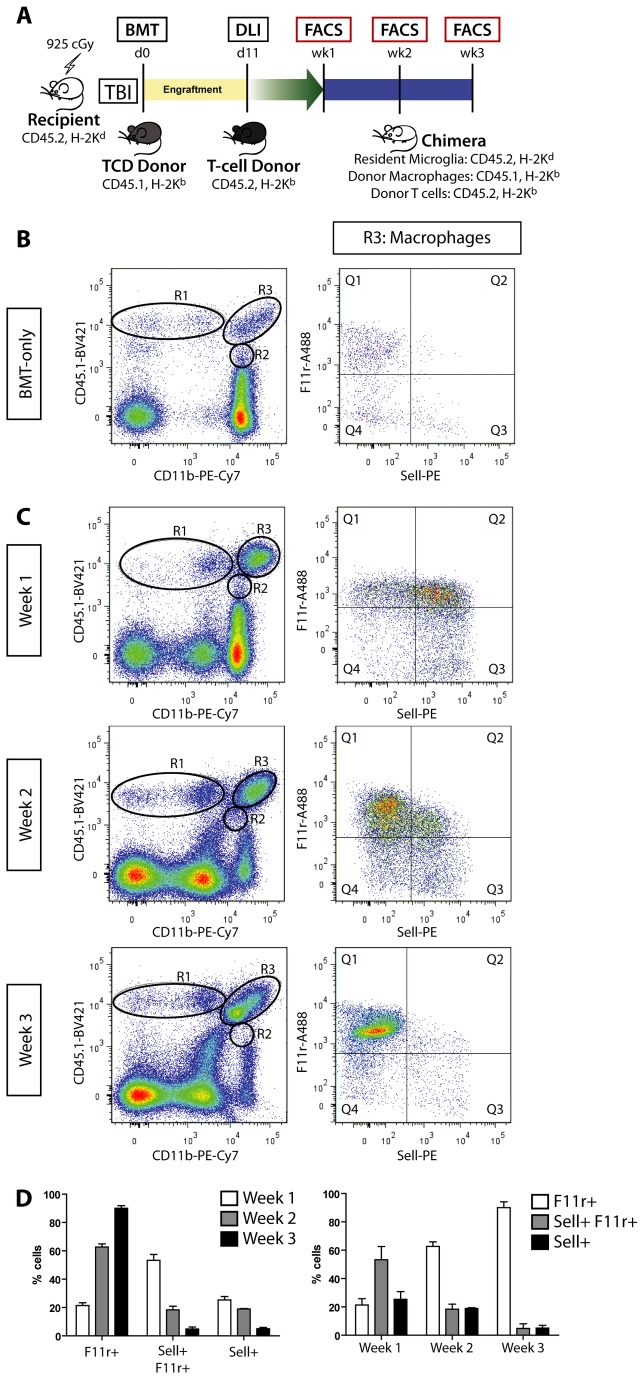
BMDM acquire F11r expression following brain infiltration. (**A**) GVHD was induced in recipient BALB/cJ mice following total body irradiation (TBI) and bone marrow transplantation (BMT) from T cell depleted (TCD) B6.SJL-*Ptprc* ^*a*^ *Pepc^b^/BoyJ* donors. Following C57BL/6J mouse donor lymphocyte infusions (DLI), immune cell infiltration was assessed by flow cytometry at 1 week, 2 weeks, and 3 weeks post-DLI (n=6 GVHD and n=6 BMT-only control per time point). (**B**) Control BMT-only mice do not exhibit GVHD and lack substantial macrophage (R3) or lymphocyte (R1, and R4 in Figure S4) infiltration. There is a very small CD11b^+^, CD45.1^high^ monocyte population that expresses F11r in the control brain (<2%; R3, right panel). (**C**) Chimeric mice with GVHD have donor monocyte infiltration (CD11b^+^, CD45.1^high^ cells; R3) with negligible CD11b^+^, CD45.1^low^ cells (R2). F11r and Sell expression (right column) of positively-labeled infiltrating donor monocytes (R3) over the course of 3 weeks of GVHD demonstrates a shift from F11r^+^ Sell^+^ cells to F11r^+^ only cells. (D) Bar graphs illustrate the mean and SEM for each population of immune system cells over the course of the three weeks following DLI.

To demonstrate that Sell^+^ monocytes acquire F11r expression as a consequence of brain infiltration, we employed allogeneic BMT to track infiltrating monocytes and resident microglia by virtue of unique CD45 antigen expression (Figure **4A**). In this model, chimeric mice with GVHD have CD45.2-expressing resident microglia and CD45.1-expressing donor BMDM and exhibit brain mononuclear infiltration following DLI, whereas control animals (BMT-only) have negligible monocyte infiltration (Figure **4B**). Resident CD45.2 microglia from both control and GVHD mice are F11r^+^ and remain F11r^+^ throughout the course of the disease (3 weeks) without increasing CD45.2 surface expression (Figure **S4**, Table **S6**) or expressing CD45.1 (Figure **4C**, CD11b^+^ CD45.1^-^ population below R2). As observed in induced murine GBM, infiltrating CD45.1^+^ BMDM (Figure **4C**, Table **3**) become F11r^+^ as a function of time after entry into the brain: in the first week post-DLI, the majority of the CD45.1^+^ infiltrating macrophages (R3) express surface Sell (53% F11r^+^ Sell^+^, 25% F11r^-^ Sell^+^); however, by the third week post-DLI, 90% of the positively-labeled R3 cells express only F11r. No other population of infiltrating cells changed their F11r and Sell expression (data not shown). Collectively, these data strongly suggest that F11r is a marker of brain macrophages in the context of murine GBM, regardless of tissue origin, prompting us to examine its prognostic value in patients with high-grade glioma.

**Table 3:** F11r and Sell surface expression of donor monocyte infiltration in chimeric mice with GVHD.

	**Week 1**	**Week 2**	**Week 3**
**%Q1: Sell- , F11r+**	21.4 +/- 2.0	62.7 +/- 2.2	90.0 +/- 1.8
**%Q2: Sell+ , F11r+**	53.3 +/- 4.2	18.3 +/- 2.6	4.9 +/- 1.4
**%Q3: Sell+ , F11r-**	25.3 +/- 2.4	19.0 +/- 0.4	5.1 +/- 0.9

F11r and Sell expression of positively-labeled infiltrating donor monocytes (CD11b^+^, CD45.1^high^ cells) over the course of 3 weeks of GVHD demonstrates a shift from 53% F11r^+^ Sell^+^ cells to 90% F11r^+^ only cells.

**Figure 5:**
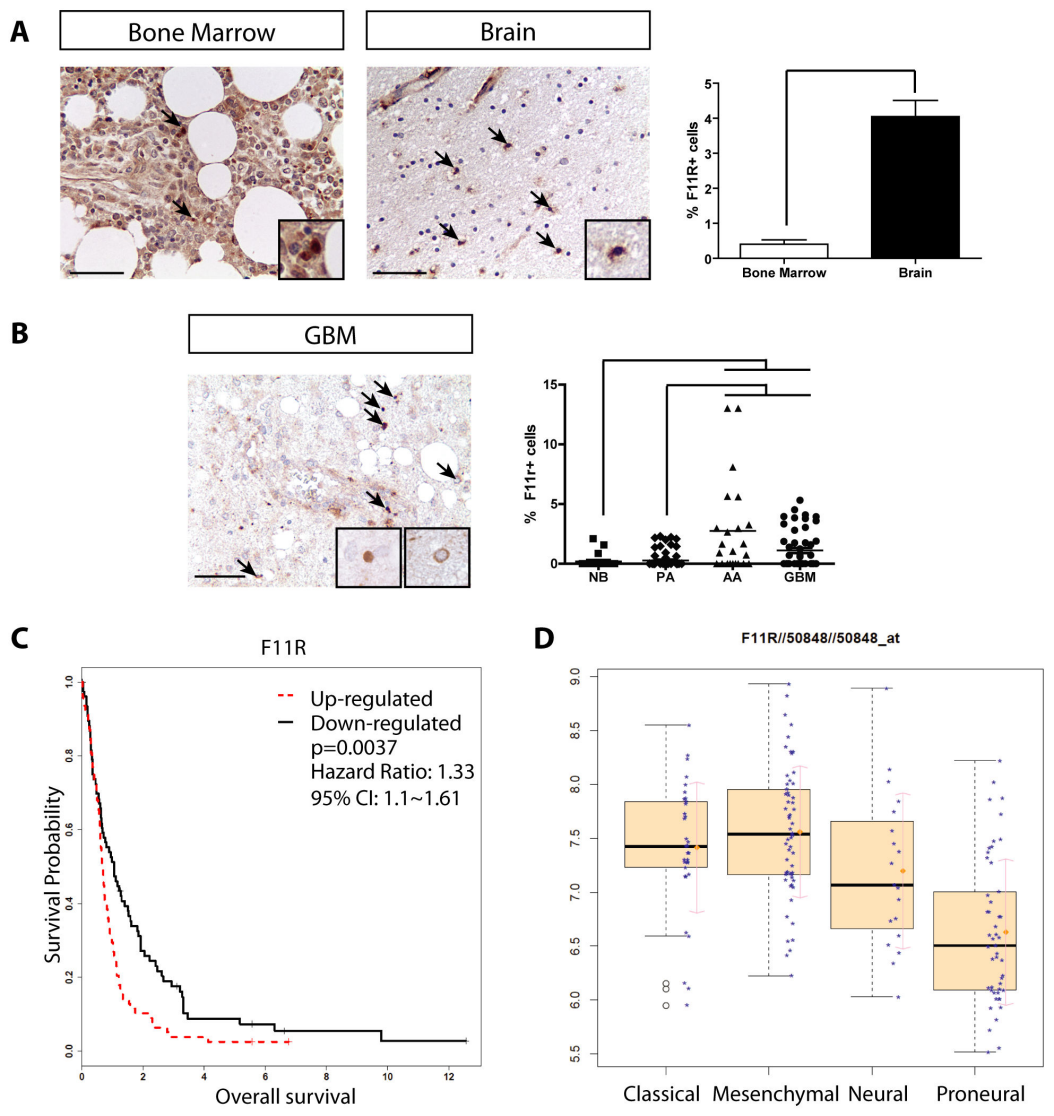
F11R expression correlates with GBM malignancy grade and survival. (**A**) Immunohistochemistry demonstrates that human bone marrow sections (n=3) have few F11R^+^ cells (arrows), while neurologically-normal post-mortem human frontal cortex brain sections contain numerous F11R^+^ mononuclear cells (n=3) (p= 0.0016). Endothelial cell labeling in the upper left quadrant of the brain section represent a positive control for staining. Scale bar = 50µm. Insets depict representative positively-labeled mononuclear cells. (**B**) A representative high-grade glioma, GBM, contains many F11R+ cells. Increased percentages of F11R^+^ cells (Kruskal-Wallis test/Dunn's Multiple Comparison Test, p<0.0001) are observed in high-grade glioma (AA, anaplastic astrocytoma, n=23; GBM, n=52) relative to low-grade tumors (PA, pilocytic astrocytoma, n=73) or normal brain (NB, n=23). (**C**) Kaplan-Meier curves and log rank test demonstrate that increased *F11R* expression negatively correlated with patient survival (GEO database: GSE16011, n=159, p=0.0037). (**D**) *F11R* was highly expressed in GSE16011 GBM samples assigned to Classical and Mesenchymal TCGA subtypes relative to the Proneural subtype (p=3.94E-12).

In human gliomas, 30-50% of the cells are CD68^+^ or IBA1^+^ tissue macrophages; however, we previously showed that the percentage of CD68^+^ or IBA1^+^ cells in these human tumors did not correlate with glioma malignancy grade [2]. To determine whether F11R expression was associated with increasing glioma malignancy grade, we first confirmed the differential protein expression of F11R in histological sections of human bone marrow and brain (p=0.0016) (Figure **5A**). Second, we quantified the percent of F11R^+^ cells (both resident and infiltrating macrophages) in a series of glioma tissue microarrays containing all glioma malignancy grades. In contrast to our prior findings with CD68 and IBA1, we found a positive correlation between the percent of F11R^+^ cells and high-grade glioma (p<0.0001) (Figure **5B**). 

Because the percent of F11R^+^ cells was greatest in the high-grade gliomas, we next asked whether F11R expression had prognostic value in predicting patient survival. Using GBM data containing 159 specimens (GSE16011) [23] dichotomized by median *F11R* expression, high *F11R* expression was associated with reduced patient survival (HR=1.33, log rank test p=0.0037) (Figure **5C**). Previous studies have demonstrated that differences in survival relate to specific GBM molecular subtypes [29], and that expression of macrophage/microglia-related genes are associated with the Mesenchymal subtype [3]. In this regard, we also found that *IBA1* (*AIF1*) and *CD68* expression was increased in Mesenchymal subtype tumors and was associated with decreased survival (Table **S7** and **S8**). However, using a multivariate Cox model to account for molecular subtype assignments, survival outcomes using *AIF1* and *CD68* were strongly influenced by the molecular subtype (continuous expression analysis: Subtype overall p=0.0065 and p= 0.0188, respectively; dichotomized expression analysis: Subtype overall p=0.0072 and p= 0.0128, respectively; Table **S9**). As a result, *CD68* was no longer prognostic for patient survival when we account for these robust subtype contributions (continuous expression analysis: HR=1.16 p=0.1222, dichotomous expression analysis: HR=1.36 p=0.0886). *AIF1* retained overall prognostic value (continuous expression analysis: HR=1.32 p=0.0155, dichotomous expression analysis: HR=1.42 p=0.0496); however, it is strongly dependent on the survival difference between the Proneural vs. Mesenchymal subtypes. While *F11R* was also more highly expressed in the Mesenchymal subtypes relative to the Proneural subtype (p = 5.74E-11) (Figure **5D**), *F11R* expression had the greatest independent prognostic value regardless of molecular subtype (Dichotomized gene expression HR=1.57, p=0.0189) (Table **4**). In contrast, the expression levels of two frequently-employed macrophage and microglia markers, CCR2 and CX3CR1, were not predictive of patient survival in GBM (Table **S8** and **S9**). Together, these data establish F11R as a novel monocyte predictor of patient outcome in GBM.

**Table 4:** Relative contributions of TCGA subtypes to the survival outcome of GBM patients dichotomized by *F11R* expression.

**Variable**	**Subtype contribution**	**P-value**	**HR and 95% CI**
**F11R: High vs. Low**		0.0189*	1.57 (1.08~2.29)
	**Subtype (overall)**	0.0787	
	**Classical vs. Mesenchymal**	0.5990	0.89 (0.57~1.39)
	**Neural vs. Mesenchymal**	0.6608	1.13 (0.66~1.95)
	**Proneural vs. Mesenchymal**	0.0239*	0.6 (0.38~0.93)

A multivariate Cox model was used to account for the contributions of specific TCGA subtypes of the GBM samples in the GSE16011 GEO dataset, and hazard ratios (HR), 95% confidence intervals (CI), and associated p-values were generated. Patients with Proneural subtype tumors had significantly increased survival compared to patients with the Mesenchymal subtype when dichotomized by F11R expression (Hazard Ratio (HR) = 0.6; p=0.0239). However, *F11R* expression demonstrates the greatest prognostic value independent of molecular subtype (multivariate Cox model; HR=1.57, p=0.0189). The full analysis is available in Table S9.

## Discussion

In the current study, we applied converging digital genomic platforms and analysis methodologies to study microglia and macrophage infiltration in GBM, yielding several important findings. First, we have created an enabling resource by generating a comprehensive catalog of differentially-expressed monocyte/microglia transcripts that may be used to investigate macrophage populations in the other CNS disease states. In addition to identifying known macrophages and microglia markers, including CCR2, LY6C, SELL, SERPINE2, SPARC, CCL4, CX3CR1, and TREM2 [30–32], we have also discovered and validated a number of novel differentially-expressed microglia and monocyte transcripts (P2RY13, CADM1, MET, CD93, and KIT). Importantly, we identified several new markers amenable to flow cytometry analysis not previously reported to be differentially expressed between microglia and BMDM (F11R, CD81, and CLEC12A). 

Second, we leveraged two representative transcripts to demonstrate that brain macrophages in murine GBM express F11R regardless of tissue origin (bone marrow versus brain). Specifically, CD11b^+^ CD45^low^ brain macrophages (microglia) and CD11b^+^ CD45^high^ infiltrating macrophages (macrophages) express F11R, rather than the SELL BMDM marker. Furthermore, using allogeneic bone marrow chimeras, we establish that BMDM entering the brain in the setting of GVHD convert from a Sell^+^ macrophage population to an F11r^+^ macrophage population as a function of time following CNS infiltration. While it is possible that Sell^+^ macrophages are eliminated and a rare population of F11r^+^ macrophages preferentially expanded, we favor a model in which F11R conversion identifies a subset of GBM-associated macrophages. In either case, the local brain environment is critical for dictating the profile change of infiltrating monocytes. The importance of monocyte F11R expression to glioma biology is further supported by the finding that more established markers of monocytes (e.g., IBA1, CD68), infiltrating macrophages (e.g., CCR2), and microglia (e.g., CX3CR1) do not provide prognostic information for patients with GBM by both immunohistological and gene expression analyses across tumor grade and molecular subtypes.

While it is tempting to postulate that F11R expression confers new biological properties for brain macrophages, the function of F11R in monocytes has not been investigated. Previous studies have shown that F11R is expressed in the basal processes of endothelial tight junctions [33] where it influences epithelial morphology and matrix adhesion [34,35], as well as immune system trafficking [36–38], and may additionally act as a leukocyte adhesion molecule to facilitate leukocyte transendothelial migration under inflammatory conditions [39]. Further studies will be required to determine whether F11R^+^ macrophages in the setting of CNS malignancy represent a unique subset of monocytes that elaborate specific chemokines and cytokines critical for GBM pathogenesis and progression.

Third, we show that one of these differentially-expressed transcripts, F11R, serves as a unique predictive biomarker for malignant glioma. Our previous studies demonstrated that the percentage of CD68^+^ and IBA1^+^ cells were increased in gliomas relative to non-neoplastic brain, with both low-grade and high-grade gliomas harboring equivalent percentages of macrophages. In contrast, we now show that the percentage of F11R^+^ macrophages are increased in high-grade relative to low-grade glioma and that F11R tumor expression is an independent predictor of patient outcome, regardless of GBM molecular subtype. Taken together with reports revealing that microglia are essential drivers of murine glioma formation and maintenance [1,2,5,8,9], along with studies in other cancer types highlighting the predictive value of stromal gene expression patterns in predicting patient outcome [40,41], the identification of F11R as a marker of a subset of brain macrophages may facilitate the detection of critical microenvironmental factors suitable for future stroma-directed glioma therapy. Similarly, potential derivative microglia/macrophage gene signatures may enable clinically-useful deconvolution of existing complex TCGA datasets for prognostic analysis.

## Supporting Information

Methods S1 **Additional details of RNA-seq and microarray analysis methods.**
(DOC)

Figure S1 **F11R staining of the different F11R+ cell types in the GBM tissue microarray.** Representative mononuclear cells (A-D), neurons (E-H), and endothelial cells (I-J) are shown. Black arrows denote positively-stained cells. Neoplastic GFAP+ astroglial cells did not express F11R (K-L), and a positive mononuclear cell is shown as an internal control for positive staining (black arrowhead). Scale bars = 10µm.
(TIF)

Figure S2 **RNA assessment of flow-sorted cells.** Three sets of BMDM and BSM samples were flow sorted for Illumina RNA-Seq (S1-S6), and two additional independent sets were generated and submitted for the Affymetrix Mouse Exon 1.0ST microarray; samples S3 and S6 were shared between the two platforms. Agilent RNA 6000 Pico results reveal minimal RNA degradation. Each sample was run in duplicate, except sample S6, due to the limitation of 11 sample wells.
(TIF)

Figure S3 **Microglia and macrophages in murine induced-glioblastoma.** Iba1 immunohistochemistry on fixed frozen sections shows increased microglia and macrophage infiltration in tumors from *Ntv-a Ink4a-Arf-/-*;Gli-luc mice injected with RCAS-PDGFB (Glioma, n=3) and matched controls (n=3). Scale bars = 100µm.
(TIF)

Figure S4 **CD45.2^+^ expressing cells in the brains of BMT and GVHD mice.** (**A**) Control BMT mice without GVHD have a main CD11b^+^ CD45.2^low^ cell population representing microglia (R5), and lack lymphocytes (R4) or macrophages (R6). The microglia are F11r^+^ only (>99%). (**B**) Chimera mice with GVHD have donor lymphocytes (R4) that are primarily CD11b^-^ CD45.2^+^ H-2K^b+^ and microglia (R5) that are CD11b^+^ CD45.2^+^ (>99%). CD45.2^high^ cells that would be denoted by R6 are not present. Microglia from GVHD mice are almost exclusively F11r^+^ (right panels) throughout the 3 weeks, similar to BMT control mice. Two independent experiments were conducted, consisting of GVHD mice (n=6) and BMT-only control mice (n=6) per time cohort.
(TIF)

Table S1 **Antibodies.**
(DOC)

Table S2 **Mouse qPCR primers.**
(DOC)

Table S3 **RNA quality assessment from flow-sorted cells.** RNA concentrations (ng/µl) were determined using the Nanodrop 1000 (Thermo Scientific) and Qubit™ (Life Technologies). For samples S2, S4, and S5, where Qubit values were below the detection limits (BL), we used RNA concentration values determined using Agilent software (0.7ng/µl, 0.04ng/µl, and 0.6ng/µl, respectively). RNA Quality Index (RQI) values were determined at the Laboratory for Clinical Genomics using the BioRad Experian assay. RNA Integrity Index (RIN) values were determined on the Agilent RNA Pico BioAnalyzer assay using 1:2 dilutions of S1, S3, and S6, and N_o_ dilutions of S2, S4, and S5.
(DOC)

Table S4 **RNA transcriptome metrics from flow-sorted cells.** Despite differences in RNA input, all samples had similar total sequence input, total reads, and reads mapped, and result in differentially expressed genes and transcripts between the bone marrow derived monocyte and brain microglia samples.
(DOC)

Table S5 **RNA-seq and microarray platforms were analyzed two ways each, with Cufflinks and ALEXA-Seq, and with Aroma and Partek, respectively.** The analyses were merged by filtering for transcripts that were significantly differentially expressed in three out of the four methods, and then were ranked by average fold change rank across the four methods.
(XLS)

Table S6 **F11r and Sell surface expression of resident microglia in chimeric mice with GVHD.** Chimera mice with GVHD have microglia that are CD11b^+^ CD45.2^+^ (>99%). Microglia from GVHD mice are almost exclusively F11r^+^ throughout the 3 weeks, similar to BMT control mice.
(DOC)

Table S7 **Gene expression levels in GBM samples (n=159).** The GSE16011 GEO dataset was stratified by TCGA subtype (Classical, n=33; Mesenchymal, n=58; Neural, n=19; and Proneural, n=49) based on the 10-nearest neighbor method. The median levels of expression of each gene were reported with interquartile range and compared using pairwise tests.
(DOC)

Table S8 **Survival outcomes of GBM patients stratified by TCGA molecular subtypes.** Gene expression levels of GBM samples within the GSE16011 GEO dataset stratified by TCGA subtype were correlated with survival outcomes using the Cox proportional hazard model to generate hazard ratios (HR), 95% confidence intervals (CI), and associated p-values. A log rank test was used to compare survival differences between the low/high expression groups (dichotomized by the median expression levels).
(DOC)

Table S9 **Relative contributions of TCGA subtypes to the survival outcome of GBM patients.** Continuous and dichotomized gene expression (high and low expression relative to the median) was analyzed using a multivariate Cox model to account for the relative contributions of specific TCGA subtypes of the GBM samples in the GSE16011 GEO dataset, and hazard ratios (HR), 95% confidence intervals (CI), and associated p-values were generated.
(DOC)
