# Supplementary material for: F11R Is a Novel Monocyte Prognostic Biomarker for Malignant Glioma
Source: PLoS One. 2013 Oct 11;8(10):e77571. doi: 10.1371/journal.pone.0077571 (PMC3795683; doi:10.1371/journal.pone.0077571)
Supplement: Methods S1 — Additional details of RNA-seq and microarray analysis methods. (DOC) [file pone.0077571.s001.doc]

**Methods S1**

***RNA-seq Cufflinks and ALEXA-Seq analysis***

Fragments Per Kilobase of exon model per Million mapped reads (amount of sequence normalized for the number of mapped reads, FPKM) values for each transcript and gene were used to generate transcript levels for comparisons both within and between samples. Further analysis was restricted to genes with an adjusted FPKM ≥ 3 (after removal of ribosomal sequence reads), which corresponds to about 0.3 transcripts per cell, to avoid concentrating on lowly-expressed mRNAs and isoforms. Clustering analysis was performed in R by hierarchical method with average linkage and Euclidian distance metric. Heat maps were generated using a customized version of heatmap.2 (gplots library) from the Cufflinks analysis.

***Microarray Partek and Aroma analysis***

After importing Affymetrix CEL files into Partek, all probes, including the control probes, were interrogated and filtered to include the full meta-probe set. Pre-background adjustments were made in the Partek workflow [1–4] for GC correction and probe sequence bias, with RMA background correction of quantile normalization on linear probe values (no log transform), and the probe set summarization was made using median polish. Differential expression was determined by a 1-way ANOVA model by using Method of Moments [5] with a log base 2 transform producing a p-value, Fold Change and Mean Ratio. Affymetrix CEL files imported into Aroma were corrected for the RMA background with quantile normalization and Log additive Probe-Level Modeling (PLM) to compensate for array artifacts and systematic biases [6]. Linear gene level expression levels were differentially compared by a two-tailed Student’s t-test and Benjamini & Hochberg false discovery rate method was applied on resulting p-values to produce q-values for multiple testing correction [7].

***Convergence of analyses*.** Ensembl gene IDs were used to converge the four independent analyses (Cufflinks, ALEXA-Seq, Aroma, and Partek). The Partek RefSeq annotation was intersected with the Ensembl v58 annotation to assign Ensembl IDs. The p-value from each analysis was adjusted to a q-value using the Benjamini and Hochberg multiple testing correction [7].

**References S1**

1. Bolstad BM, Irizarry RA, Astrand M, Speed TP (2003) A comparison of normalization methods for high density oligonucleotide array data based on variance and bias. Bioinformatics 19: 185–193.

2. Irizarry RA, Bolstad BM, Collin F, Cope LM, Hobbs B, et al. (2003) Summaries of Affymetrix GeneChip probe level data. Nucleic Acids Res 31: e15. doi: 10.1093/nar/gng015.

3. Irizarry RA, Hobbs B, Collin F, Beazer-Barclay YD, Antonellis KJ, et al. (2003) Exploration, normalization, and summaries of high density oligonucleotide array probe level data. Biostatistics 4: 249–264. doi: 10.1093/biostatistics/4.2.249.

4. Wu Z, Irizarry RA, Gentleman R, Martinez-Murillo F, Spencer F (2004) A Model-Based Background Adjustment for Oligonucleotide Expression Arrays. J Am Stat Assoc 99: 909–917. doi: 10.1198/016214504000000683.

5. Eisenhart C (1947) The assumptions underlying the analysis of variance. Biometrics 3: 1–21.

6. Bengtsson H, Simpson K, Bullard J, Hansen K (2008) aroma.affymetrix: A generic framework in R for analyzing small to very large Affymetrix data sets in bounded memory, Tech Report #745. Department of Statistics, University of California, Berkeley,: 1–9.

7. Benjamini Y, Hochberg Y (1995) Controlling the False Discovery Rate: A Practical and Powerful Approach to Multiple Testing. Journal of the Royal Statistical Society B 57: 289–300.
