# Supplementary figures and images for: F11R Is a Novel Monocyte Prognostic Biomarker for Malignant Glioma
Source: PLoS One. 2013 Oct 11;8(10):e77571. doi: 10.1371/journal.pone.0077571 (PMC3795683; doi:10.1371/journal.pone.0077571)

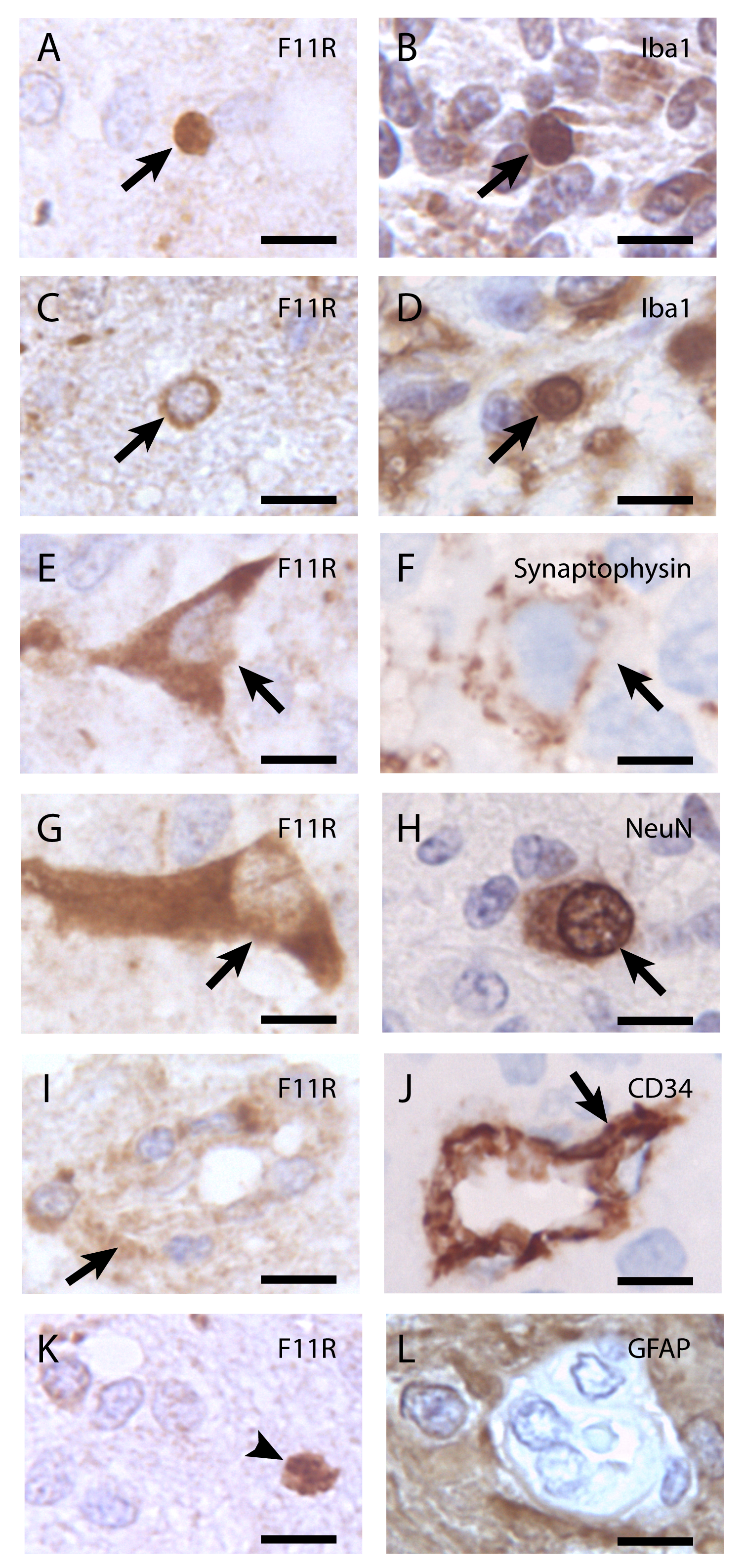

Supplement: Figure S1 — F11R staining of the different F11R+ cell types in the GBM tissue microarray. Representative mononuclear cells (A-D), neurons (E-H), and endothelial cells (I-J) are shown. Black arrows denote positively-stained cells. Neoplastic GFAP+ astroglial cells did not express F11R (K-L), and a positive mononuclear cell is shown as an internal control for positive staining (black arrowhead). Scale bars = 10µm. (TIF) [file pone.0077571.s002.tif]

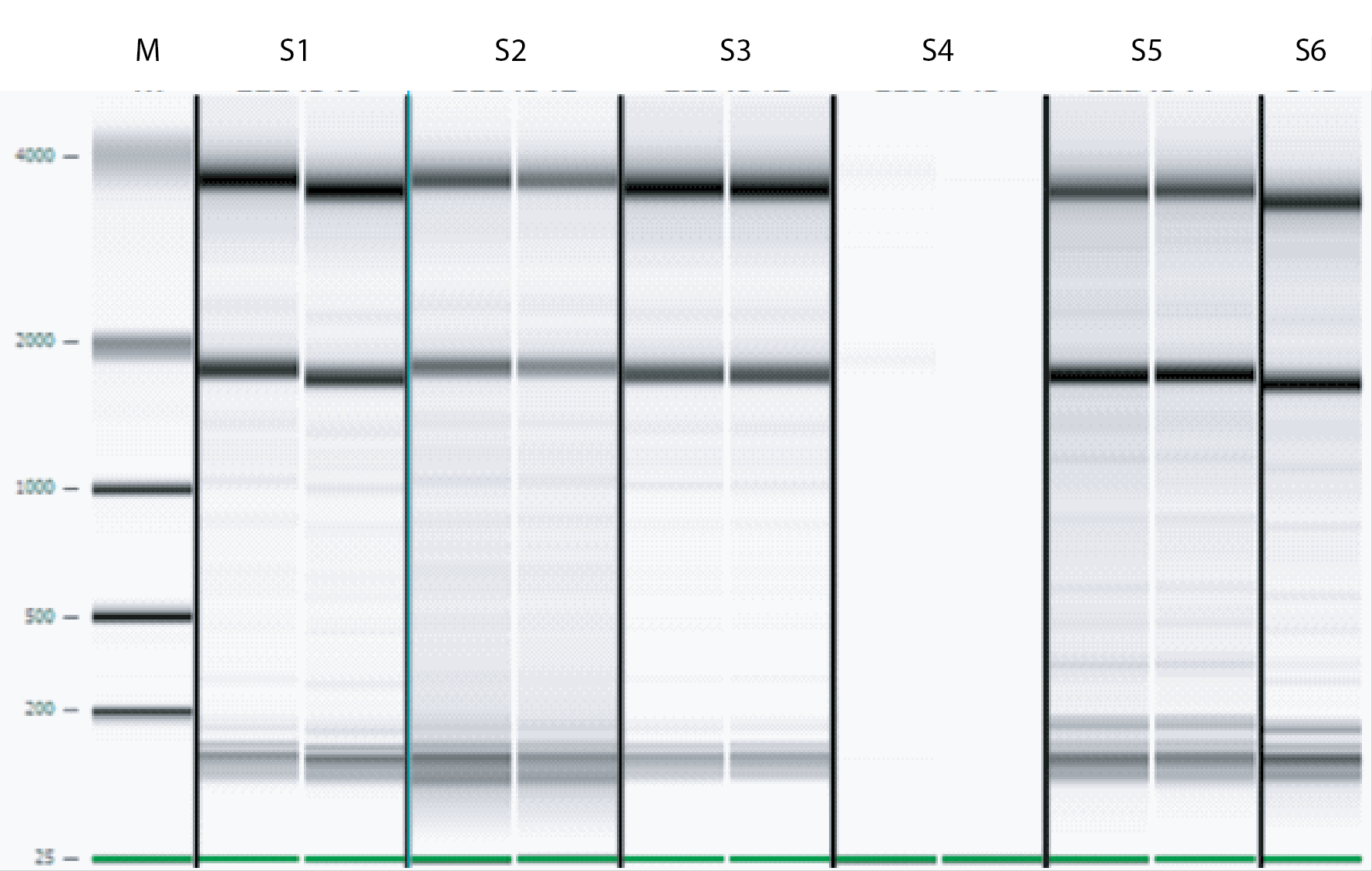

Supplement: Figure S2 — RNA assessment of flow-sorted cells. Three sets of BMDM and BSM samples were flow sorted for Illumina RNA-Seq (S1-S6), and two additional independent sets were generated and submitted for the Affymetrix Mouse Exon 1.0ST microarray; samples S3 and S6 were shared between the two platforms. Agilent RNA 6000 Pico results reveal minimal RNA degradation. Each sample was run in duplicate, except sample S6, due to the limitation of 11 sample wells. (TIF) [file pone.0077571.s003.tif]

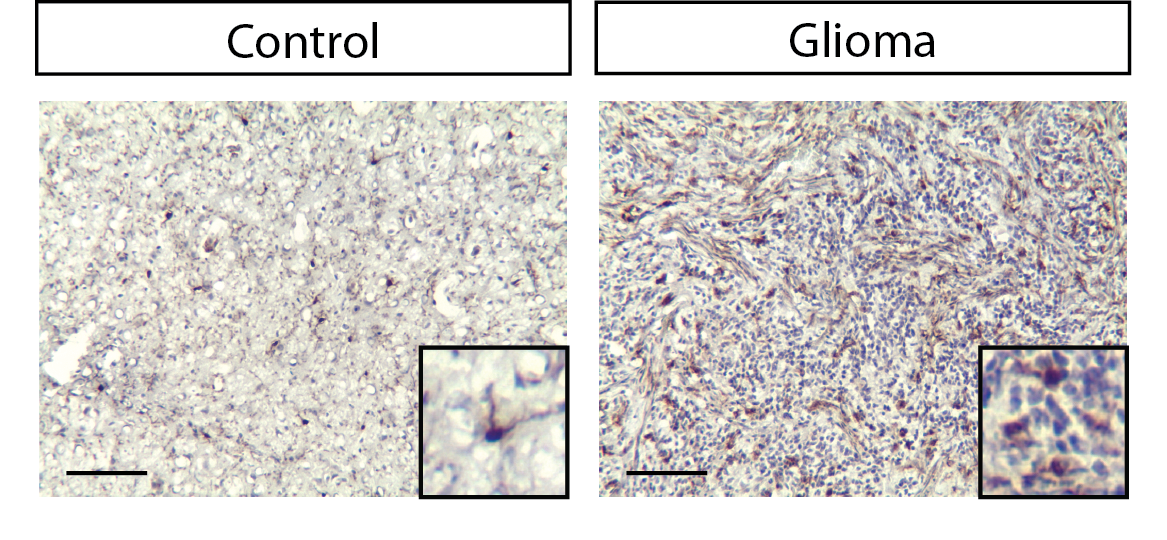

Supplement: Figure S3 — Microglia and macrophages in murine induced-glioblastoma. Iba1 immunohistochemistry on fixed frozen sections shows increased microglia and macrophage infiltration in tumors from Ntv-a Ink4a-Arf-/-;Gli-luc mice injected with RCAS-PDGFB (Glioma, n=3) and matched controls (n=3). Scale bars = 100µm. (TIF) [file pone.0077571.s004.tif]

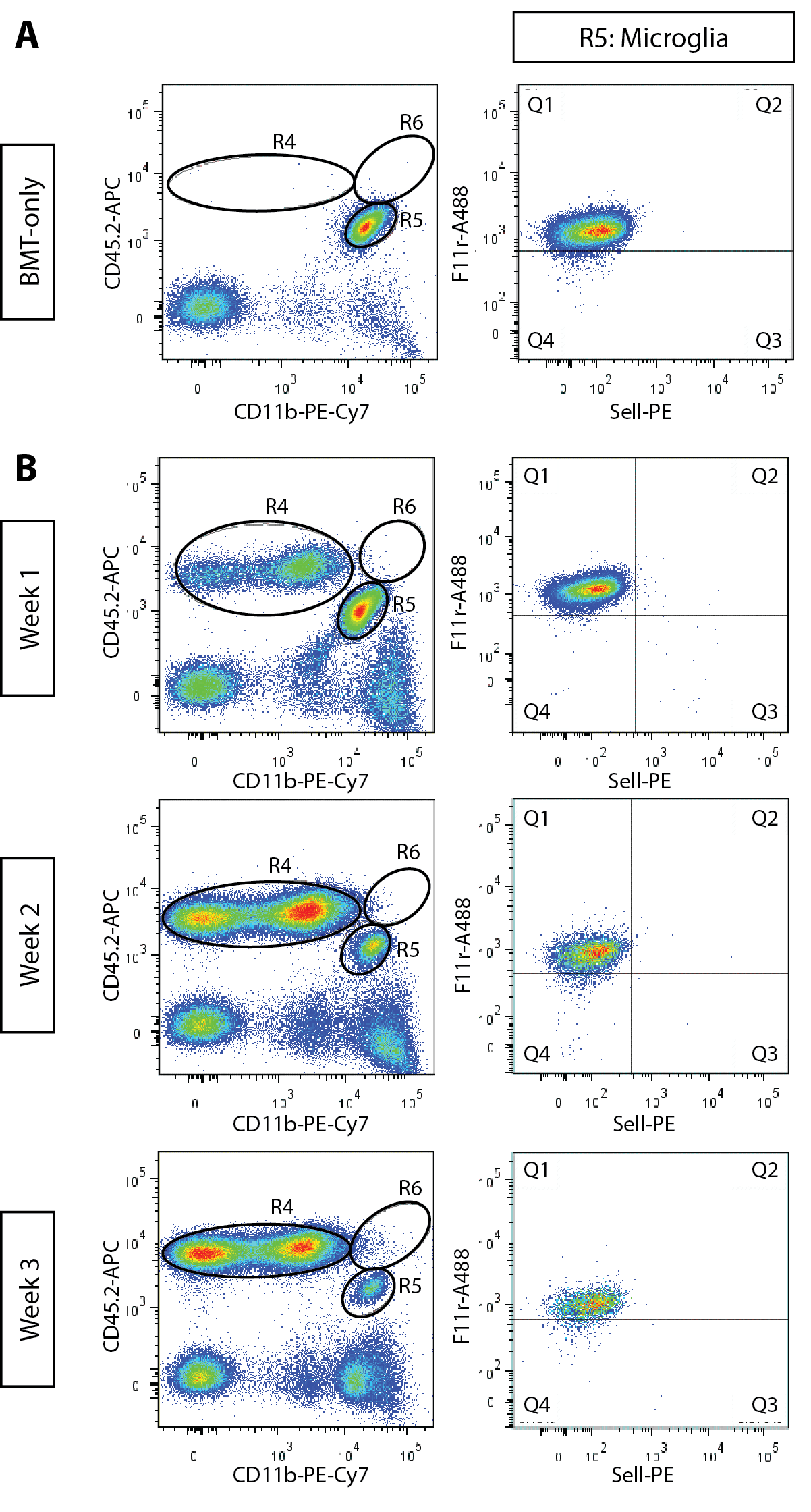

Supplement: Figure S4 — CD45.2+ expressing cells in the brains of BMT and GVHD mice. (A) Control BMT mice without GVHD have a main CD11b+ CD45.2low cell population representing microglia (R5), and lack lymphocytes (R4) or macrophages (R6). The microglia are F11r+ only (>99%). (B) Chimera mice with GVHD have donor lymphocytes (R4) that are primarily CD11b- CD45.2+ H-2Kb+ and microglia (R5) that are CD11b+ CD45.2+ (>99%). CD45.2high cells that would be denoted by R6 are not present. Microglia from GVHD mice are almost exclusively F11r+ (right panels) throughout the 3 weeks, similar to BMT control mice. Two independent experiments were conducted, consisting of GVHD mice (n=6) and BMT-only control mice (n=6) per time cohort. (TIF) [file pone.0077571.s005.tif]
