# Supplementary material for: F11R Is a Novel Monocyte Prognostic Biomarker for Malignant Glioma
Source: PLoS One. 2013 Oct 11;8(10):e77571. doi: 10.1371/journal.pone.0077571 (PMC3795683; doi:10.1371/journal.pone.0077571)
Supplement: Table S1 — Antibodies. (DOC) [file pone.0077571.s006.doc]

**Table S1.** Antibodies

| **Antigen (clone)** | **Host** | **Reactivity** | **Usage^a^** | **Source^b^** | **Dilution** |
| --- | --- | --- | --- | --- | --- |
| **CD115 (AFS98)** | Rat | Mouse | FC (APC conjugate) | BioLegend | 1:100 |
| **CD115 (AFS98)** | Rat | Mouse | FC (PE conjugate) | BioLegend | 1:100 |
| **CD11b (M1/70)** | Rat | Mouse | FC (PerCP-Cy5.5 conjugate) | BD Biosciences | 1:100 |
| **CD11b (M1/70)** | Rat | Mouse | FC (PE-Cy7 conjugate) | BioLegend | 1:100 |
| **CD16/32** | Rat | Mouse | FC | BD Biosciences | 1:50 |
| **CD45 (30-F11)** | Rat | Mouse | FC (APC-Cy7 conjugate) | BioLegend | 1:100 |
| **CD45 (30-F11)** | Rat | Mouse | FC (APC conjugate) | BD Biosciences | 1:100 |
| **CD45.1 (A20)** | Mouse | Mouse | FC (BV421 conjugate) | BioLegend | 1:25 |
| **CD45.2 (104)** | Mouse | Mouse | FC (APC conjugate) | BioLegend | 1:100 |
| **CD81 (Eat-2)** | Armenian Hamster | Mouse | FC (PE conjugate) | BioLegend | 1:100 |
| **Clec12A** | Goat | Mouse | FC (PE conjugate) | R&D Systems | 1:10 |
| **F11R (2E3-1C8)** | Mouse | Human | IHC (Citrate retrieval) | Abnova | 1:500 |
| **F11r (H202-106)** | Rat | Mouse | FC (A488 conjugate) | AbD Serotec | 1:100 |
| **H-2K^b^ (AF6-88.5)** | Mouse | Mouse | FC (PerCP-Cy5.5 conjugate) | BioLegend | 1:25 |
| **Iba1** | Rabbit | Mouse | IHC | Wako | 1:2000 |
| **Ly6G (1A8)** | Rat | Mouse | FC (APC-Cy7 conjugate) | BioLegend | 1:100 |
| **Ly6G (1A8)** | Rat | Mouse | FC (BV421 conjugate) | BioLegend | 1:25 |
| **Ly6G (1A8)** | Rat | Mouse | FC (FITC conjugate) | BioLegend | 1:200 |
| **Sell (MEL-14)** | Rat | Mouse | FC (PE conjugate) | BioLegend | 1:100 |
| **Sell (MEL-14)** | Rat | Mouse | FC (PE-Cy7 conjugate) | BioLegend | 1:100 |
| **Armenian Hamster IgG Isotype** | Armenian Hamster | Mouse | FC (PE conjugate) | BioLegend | 1:100 |
| **Normal Goat IgG Isotype** | Goat | Mouse | FC (PE conjugate) | R&D Systems | 1:10 |
| **Rat IgG1 isotype** | Rat | Mouse | FC (A488 conjugate) | AbD Serotec | 1:100 |
| **Rat IgG2a, κ isotype (RTK2758)** | Rat | Mouse | FC (PE conjugate) | BioLegend | 1:100 |

^a^ FC (flow cytometry), IF (immunofluorescence), IHC (immunohistochemistry)

**^b^** AbD Serotec, Kidlington, Oxford, UK; Abnova, Neihu District. Taipei City, Taiwan; BD Biosciences, San Jose, CA; BioLegend, San Diego, CA; R&D Systems, Minneapolis, MN; Wako Pure Chemical Industries, Ltd., Osaka, Japan
