# Supplementary material for: F11R Is a Novel Monocyte Prognostic Biomarker for Malignant Glioma
Source: PLoS One. 2013 Oct 11;8(10):e77571. doi: 10.1371/journal.pone.0077571 (PMC3795683; doi:10.1371/journal.pone.0077571)
Supplement: Table S2 — Mouse qPCR primers. (DOC) [file pone.0077571.s007.doc]

**Table S2.** Mouse qPCR primers

| **Gene** | **Forward Primer** | **Reverse Primer** |
| --- | --- | --- |
| ***B2m (house-keeping)*** | TaqMan Gene Assay: Mm00437762_m1 | |
| ***Cadm1*** | 5’- ACCACCATCACAGTCCTGGTTCCT -3’ | 5’- TGCTGGCCATGGCAGTACAGTTG -3’ |
| ***Cd81*** | 5’- CCCAACACCTTCTACGTGGGCATC -3’ | 5’- GATGGCCCCATAGCACCCCA -3’ |
| ***Cd93*** | TaqMan Gene Assay: Mm00440239_g1 | |
| ***Clec12a*** | 5’- TCGGAATGCCAGCCTCCTGAAG -3’ | 5’- GTGTGCGATCTTTTCTGGGCAACA -3’ |
| ***F11r*** | 5’- GTGCTTGTACCTCCATCCAAGCCG -3’ | 5’- GAGGGTGGGGAACCATCATGCTC -3’ |
| ***H3f3a (house-keeping)*** | 5’- CGTGAAATCAGACGCTATCAGAA -3’ | 5’- TCGCACCAGACGCTGAAAG -3’ |
| ***Kit*** | TaqMan Gene Assay: Mm00445212_m1 | |
| ***Mertk*** | 5’- GCCCTCCTGAGCCCGTCAATATC -3’ | 5’- TGTCTCTGTCAGACCAGGTACGGT -3’ |
| ***Met*** | TaqMan Gene Assay: Mm01156972_m1 | |
| ***P2ry13*** | 5’- AGCAGGATCAAGCGAAGACCACC -3’ | 5’- AGACGTGAGGCCATGCGTGA -3’ |
| ***Sell*** | TaqMan Gene Assay: Mm00441291_m1 | |
