# Supplementary material for: F11R Is a Novel Monocyte Prognostic Biomarker for Malignant Glioma
Source: PLoS One. 2013 Oct 11;8(10):e77571. doi: 10.1371/journal.pone.0077571 (PMC3795683; doi:10.1371/journal.pone.0077571)
Supplement: Table S3 — RNA quality assessment from flow-sorted cells. RNA concentrations (ng/µl) were determined using the Nanodrop 1000 (Thermo Scientific) and Qubit™ (Life Technologies). For samples S2, S4, and S5, where Qubit values were below the detection limits (BL), we used RNA concentration values determined using Agilent software (0.7ng/µl, 0.04ng/µl, and 0.6ng/µl, respectively). RNA Quality Index (RQI) values were determined at the Laboratory for Clinical Genomics using the BioRad Experian assay. RNA Integrity Index (RIN) values were determined on the Agilent RNA Pico BioAnalyzer assay using 1:2 dilutions of S1, S3, and S6, and No dilutions of S2, S4, and S5. (DOC) [file pone.0077571.s008.doc]

- **Table S3. RNA quality assessment from flow-sorted cells.** RNA concentrations (ng/µl) were determined using the Nanodrop 1000 (Thermo Scientific) and Qubit™ (Life Technologies). For samples S2, S4, and S5, where Qubit values were below the detection limits (BL), we used RNA concentration values determined using Agilent software (0.7ng/µl, 0.04ng/µl, and 0.6ng/µl, respectively). RNA Quality Index (RQI) values were determined at the Laboratory for Clinical Genomics using the BioRad Experian assay. RNA Integrity Index (RIN) values were determined on the Agilent RNA Pico BioAnalyzer assay using 1:2 dilutions of S1, S3, and S6, and N_o_ dilutions of S2, S4, and S5.

| **Sample ID** | **Cell #** | **Cell Type** | **Gender** | **Nanodrop**  **(ng/µl)** | **Qubit**  **(ng/µl)** | **Experion**  **RQI** | **Agilent**  **RIN** |
| --- | --- | --- | --- | --- | --- | --- | --- |
| **S1** | 112,000 | Monocyte | M | 15 | 9.2 | 6.2 | 8.6 |
| **S2** | 23,233 | Monocyte | F | 5 | BL (0.7) | 7.9 | 7.8 |
| **S3** | 99,153 | Monocyte | F | 17 | 11.7 | 9.0 | 9.0 |
| **S4** | 218,718 | Microglia | M | 2 | BL (0.04) | n/a | 0.3 |
| **S5** | 125,711 | Microglia | F | 8 | BL (0.6) | 7.0 | 7.6 |
| **S6** | 254,908 | Microglia | F | 13 | 7.7 | 7.9 | 8.0 |
