# Supplementary material for: F11R Is a Novel Monocyte Prognostic Biomarker for Malignant Glioma
Source: PLoS One. 2013 Oct 11;8(10):e77571. doi: 10.1371/journal.pone.0077571 (PMC3795683; doi:10.1371/journal.pone.0077571)
Supplement: Table S4 — RNA transcriptome metrics from flow-sorted cells. Despite differences in RNA input, all samples had similar total sequence input, total reads, and reads mapped, and result in differentially expressed genes and transcripts between the bone marrow derived monocyte and brain microglia samples. (DOC) [file pone.0077571.s009.doc]

**Table S4. RNA transcriptome metrics from flow-sorted cells.** Despite differences in RNA input, all samples had similar total sequence input, total reads, and reads mapped, and result in differentially expressed genes and transcripts between the bone marrow derived monocyte and brain microglia samples.

| **Sample ID** | **Total Sequence Gbp** | **Total Reads** | **Reads Mapped** | **% Mapped** | **Genes Observed (Cufflinks FPKM >= 1)** | **Transcripts Observed (Cufflinks FPKM >= 1)** |
| --- | --- | --- | --- | --- | --- | --- |
| **S1** | 15.82 | 158,240,128 | 100,425,066 | 63% | 7282 | 10092 |
| **S2** | 19.76 | 197,627,558 | 90,258,964 | 46% | 7910 | 11202 |
| **S3** | 17.00 | 169,992,646 | 109,419,795 | 64% | 7704 | 10875 |
| **S4** | 22.66 | 226,570,788 | 127,169,557 | 56% | 5161 | 6780 |
| **S5** | 16.07 | 160,667,138 | 100,544,148 | 63% | 6531 | 8370 |
| **S6** | 17.96 | 179,586,516 | 116,445,417 | 65% | 6348 | 8147 |
