# Supplementary material for: F11R Is a Novel Monocyte Prognostic Biomarker for Malignant Glioma
Source: PLoS One. 2013 Oct 11;8(10):e77571. doi: 10.1371/journal.pone.0077571 (PMC3795683; doi:10.1371/journal.pone.0077571)
Supplement: Table S6 — F11r and Sell surface expression of resident microglia in chimeric mice with GVHD. Chimera mice with GVHD have microglia that are CD11b+ CD45.2+ (>99%). Microglia from GVHD mice are almost exclusively F11r+ throughout the 3 weeks, similar to BMT control mice. (DOC) [file pone.0077571.s011.doc]

**Table S6. F11r and Sell surface expression of resident microglia in chimeric mice with GVHD.** Chimera mice with GVHD have microglia that are CD11b^+^ CD45.2^+^ (>99%). Microglia from GVHD mice are almost exclusively F11r^+^ throughout the 3 weeks, similar to BMT control mice.

|  | **Week 1** | **Week 2** | **Week 3** |
| --- | --- | --- | --- |
| **%Q1: Sell- , F11r+** | 99.9 +/- 0.0 | 99.8 +/- 0.0 | 99.8 +/- 0.0 |
| **%Q2: Sell+ , F11r+** | 0.1 +/- 0.0 | 0.2 +/- 0.0 | 0.2 +/- 0.0 |
| **%Q3: Sell+ , F11r-** | 0.1 +/- 0.0 | <0.1 | 0.1 +/- 0.0 |
