# Supplementary material for: F11R Is a Novel Monocyte Prognostic Biomarker for Malignant Glioma
Source: PLoS One. 2013 Oct 11;8(10):e77571. doi: 10.1371/journal.pone.0077571 (PMC3795683; doi:10.1371/journal.pone.0077571)
Supplement: Table S7 — Gene expression levels in GBM samples (n=159). The GSE16011 GEO dataset was stratified by TCGA subtype (Classical, n=33; Mesenchymal, n=58; Neural, n=19; and Proneural, n=49) based on the 10-nearest neighbor method. The median levels of expression of each gene were reported with interquartile range and compared using pairwise tests. (DOC) [file pone.0077571.s012.doc]

**Table S7.** **Gene expression levels in GBM samples (n=159).** The GSE16011 GEO dataset was stratified by TCGA subtype (Classical, n=33; Mesenchymal, n=58; Neural, n=19; and Proneural, n=49) based on the 10-nearest neighbor method. The median levels of expression of each gene were reported with interquartile range and compared using pairwise tests.

| **Category** | **Gene** | **Levels** | **Median**  **(Interquartile Range)** | **Pairwise tests** | **p-value** |
| --- | --- | --- | --- | --- | --- |
| ***General macrophage markers*** | AIF1 | Subtype: Overall | 9.238 (8.574-9.796) | Overall | 1.68E-10* |
|  |  | Classical | 8.56 (8.317-9.193) | Classical vs. Mesenchymal | 1.66E-10* |
|  |  | Mesenchymal | 9.818 (9.26-10.317) | Classical vs. Neural | 0.077 |
|  |  | Neural | 9.237 (8.673-9.476) | Classical vs. Proneural | 0.06 |
|  |  | Proneural | 9.043 (8.503-9.632) | Mesenchymal vs. Neural | 0.0003* |
|  |  |  |  | Mesenchymal vs. Proneural | 1.60E-07* |
|  |  |  |  | Neural vs. Proneural | 0.752 |
|  | CD68 | Subtype: Overall | 7.56 (6.923-8.248) | Overall | 3.94E-12* |
|  |  | Classical | 7.061 (6.511-7.809) | Classical vs. Mesenchymal | 4.97E-09* |
|  |  | Mesenchymal | 8.276 (7.652-8.814) | Classical vs. Neural | 0.652 |
|  |  | Neural | 7.126 (6.642-7.746) | Classical vs. Proneural | 0.844 |
|  |  | Proneural | 7.109 (6.491-7.83) | Mesenchymal vs. Neural | 8.04E-06* |
|  |  |  |  | Mesenchymal vs. Proneural | 2.55E-11* |
|  |  |  |  | Neural vs. Proneural | 0.519 |
| ***Bone marrow monocyte markers*** | SELL | Subtype: Overall | 6.322 (5.654-7.186) | Overall | 7.42E-06* |
|  |  | Classical | 5.705 (5.145-6.307) | Classical vs. Mesenchymal | 6.62E-07* |
|  |  | Mesenchymal | 6.998 (6.204-7.599) | Classical vs. Neural | 0.002* |
|  |  | Neural | 6.366 (6.134-7.141) | Classical vs. Proneural | 0.014* |
|  |  | Proneural | 6.1 (5.168-6.995) | Mesenchymal vs. Neural | 0.438 |
|  |  |  |  | Mesenchymal vs. Proneural | 0.004* |
|  |  |  |  | Neural vs. Proneural | 0.181 |
|  | MET | Subtype: Overall | 5.339 (4.992-6.063) | Overall | 9.69E-04* |
|  |  | Classical | 5.073 (4.855-5.512) | Classical vs. Mesenchymal | 0.0002* |
|  |  | Mesenchymal | 5.941 (5.339-6.605) | Classical vs. Neural | 0.499 |
|  |  | Neural | 5.351 (5.125-5.624) | Classical vs. Proneural | 0.166 |
|  |  | Proneural | 5.167 (4.928-5.565) | Mesenchymal vs. Neural | 0.017* |
|  |  |  |  | Mesenchymal vs. Proneural | 0.008* |
|  |  |  |  | Neural vs. Proneural | 0.663 |
|  | CCR2 | Subtype: Overall | 4.597 (4.502-4.829) | Overall | 9.54E-04* |
|  |  | Classical | 4.578 (4.501-4.642) | Classical vs. Mesenchymal | 0.0008* |
|  |  | Mesenchymal | 4.76 (4.576-5.065) | Classical vs. Neural | 0.962 |
|  |  | Neural | 4.585 (4.482-4.709) | Classical vs. Proneural | 0.539 |
|  |  | Proneural | 4.546 (4.441-4.642) | Mesenchymal vs. Neural | 0.006* |
|  |  |  |  | Mesenchymal vs. Proneural | 0.002* |
|  |  |  |  | Neural vs. Proneural | 0.645 |
|  | CD93 | Subtype: Overall | 9.301 (8.568-9.913) | Overall | 5.36E-14* |
|  |  | Classical | 9.181 (8.561-9.537) | Classical vs. Mesenchymal | 1.89E-05* |
|  |  | Mesenchymal | 9.93 (9.506-10.204) | Classical vs. Neural | 0.0004* |
|  |  | Neural | 8.517 (8.001-9.006) | Classical vs. Proneural | 0.086 |
|  |  | Proneural | 8.785 (8.247-9.325) | Mesenchymal vs. Neural | 2.54E-12* |
|  |  |  |  | Mesenchymal vs. Proneural | 8.73E-11* |
|  |  |  |  | Neural vs. Proneural | 0.016* |
|  | KIT | Subtype: Overall | 6.451 (5.825-7.556) | Overall | 1.42E-07* |
|  |  | Classical | 6.352 (5.92-7.581) | Classical vs. Mesenchymal | 0.052 |
|  |  | Mesenchymal | 6.034 (5.581-6.495) | Classical vs. Neural | 0.016* |
|  |  | Neural | 7.637 (6.802-8.088) | Classical vs. Proneural | 0.004* |
|  |  | Proneural | 7.282 (6.101-8.274) | Mesenchymal vs. Neural | 3.30E-05* |
|  |  |  |  | Mesenchymal vs. Proneural | 8.68E-08* |
|  |  |  |  | Neural vs. Proneural | 0.882 |
|  | CLEC12A | Subtype: Overall | 4.388 (4.288-4.622) | Overall | 3.06E-03* |
|  |  | Classical | 4.349 (4.252-4.504) | Classical vs. Mesenchymal | 0.002* |
|  |  | Mesenchymal | 4.567 (4.34-5.06) | Classical vs. Neural | 0.283 |
|  |  | Neural | 4.323 (4.272-4.448) | Classical vs. Proneural | 0.718 |
|  |  | Proneural | 4.349 (4.267-4.474) | Mesenchymal vs. Neural | 0.144 |
|  |  |  |  | Mesenchymal vs. Proneural | 0.002* |
|  |  |  |  | Neural vs. Proneural | 0.398 |
| ***Brainstem microglia markers*** | MERTK | Subtype: Overall | 7.93 (7.562-8.479) | Overall | 8.48E-05* |
|  |  | Classical | 7.637 (7.478-7.975) | Classical vs. Mesenchymal | 0.0006* |
|  |  | Mesenchymal | 8.243 (7.854-8.762) | Classical vs. Neural | 0.213 |
|  |  | Neural | 7.941 (7.848-8.151) | Classical vs. Proneural | 0.712 |
|  |  | Proneural | 7.8 (7.284-8.213) | Mesenchymal vs. Neural | 0.125 |
|  |  |  |  | Mesenchymal vs. Proneural | 2.12E-05* |
|  |  |  |  | Neural vs. Proneural | 0.103 |
|  | F11R | Subtype: Overall | 7.276 (6.594-7.772) | Overall | 5.74E-11* |
|  |  | Classical | 7.426 (7.231-7.842) | Classical vs. Mesenchymal | 0.304 |
|  |  | Mesenchymal | 7.54 (7.164-7.947) | Classical vs. Neural | 0.245 |
|  |  | Neural | 7.067 (6.664-7.657) | Classical vs. Proneural | 2.57E-07* |
|  |  | Proneural | 6.506 (6.092-7.005) | Mesenchymal vs. Neural | 0.035* |
|  |  |  |  | Mesenchymal vs. Proneural | 7.48E-12* |
|  |  |  |  | Neural vs. Proneural | 0.001* |
|  | P2RY13 | Subtype: Overall | 6.588 (5.542-7.465) | Overall | 0.074 |
|  |  | Classical | 5.975 (5.464-6.925) | Classical vs. Mesenchymal | 0.012* |
|  |  | Mesenchymal | 6.876 (5.812-7.761) | Classical vs. Neural | 0.101 |
|  |  | Neural | 6.598 (6.066-7.221) | Classical vs. Proneural | 0.246 |
|  |  | Proneural | 6.304 (5.26-7.598) | Mesenchymal vs. Neural | 0.769 |
|  |  |  |  | Mesenchymal vs. Proneural | 0.137 |
|  |  |  |  | Neural vs. Proneural | 0.433 |
|  | CADM1 | Subtype: Overall | 9.498 (8.903-9.999) | Overall | 1.81E-04* |
|  |  | Classical | 9.81 (9.371-9.96) | Classical vs. Mesenchymal | 0.337 |
|  |  | Mesenchymal | 9.476 (8.992-10.077) | Classical vs. Neural | 0.875 |
|  |  | Neural | 9.833 (9.684-10.014) | Classical vs. Proneural | 0.0002* |
|  |  | Proneural | 8.9 (8.456-9.639) | Mesenchymal vs. Neural | 0.336 |
|  |  |  |  | Mesenchymal vs. Proneural | 0.0009* |
|  |  |  |  | Neural vs. Proneural | 0.001* |
|  | CD81 | Subtype: Overall | 12.28 (11.99-12.619) | Overall | 0.374 |
|  |  | Classical | 12.281 (12.053-12.603) | Classical vs. Mesenchymal | 0.578 |
|  |  | Mesenchymal | 12.414 (11.998-12.673) | Classical vs. Neural | 0.647 |
|  |  | Neural | 12.4 (11.993-12.539) | Classical vs. Proneural | 0.353 |
|  |  | Proneural | 12.182 (11.919-12.48) | Mesenchymal vs. Neural | 0.338 |
|  |  |  |  | Mesenchymal vs. Proneural | 0.09 |
|  |  |  |  | Neural vs. Proneural | 0.775 |
|  | CX3CR1 | Subtype: Overall | 10.617 (9.484-11.496) | Overall | 0.351 |
|  |  | Classical | 10.52 (8.94-11.056) | Classical vs. Mesenchymal | 0.257 |
|  |  | Mesenchymal | 10.776 (9.758-11.62) | Classical vs. Neural | 0.164 |
|  |  | Neural | 10.673 (10.493-11.092) | Classical vs. Proneural | 0.918 |
|  |  | Proneural | 10.269 (8.722-12.034) | Mesenchymal vs. Neural | 0.56 |
|  |  |  |  | Mesenchymal vs. Proneural | 0.248 |
|  |  |  |  | Neural vs. Proneural | 0.162 |
