# Supplementary material for: F11R Is a Novel Monocyte Prognostic Biomarker for Malignant Glioma
Source: PLoS One. 2013 Oct 11;8(10):e77571. doi: 10.1371/journal.pone.0077571 (PMC3795683; doi:10.1371/journal.pone.0077571)
Supplement: Table S8 — Survival outcomes of GBM patients stratified by TCGA molecular subtypes. Gene expression levels of GBM samples within the GSE16011 GEO dataset stratified by TCGA subtype were correlated with survival outcomes using the Cox proportional hazard model to generate hazard ratios (HR), 95% confidence intervals (CI), and associated p-values. A log rank test was used to compare survival differences between the low/high expression groups (dichotomized by the median expression levels). (DOC) [file pone.0077571.s013.doc]

**Table S8. Survival outcomes of GBM patients stratified by TCGA molecular subtypes.** Gene expression levels of GBM samples within the GSE16011 GEO dataset stratified by TCGA subtype were correlated with survival outcomes using the Cox proportional hazard model to generate hazard ratios (HR), 95% confidence intervals (CI), and associated p-values. A log rank test was used to compare survival differences between the low/high expression groups (dichotomized by the median expression levels).

| **Category** | **Gene** | **Subtype** | **HR and 95% CI** | **Cox Proportional Hazard p-value** | **Log Rank Test**  **p-value** |
| --- | --- | --- | --- | --- | --- |
| ***General macrophage markers*** | AIF1 | all | 1.28 (1.05~1.55) | 0.0126* | 0.0289* |
|  |  | Classical | 1.63 (0.82~3.22) | 0.1601 | 0.1170 |
|  |  | Mesenchymal | 1.57 (1.06~2.32) | 0.0252* | 0.3400 |
|  |  | Neural | 2.34 (0.96~5.72) | 0.0627 | 0.2936 |
|  |  | Proneural | 1.06 (0.75~1.5) | 0.7465 | 0.8808 |
|  | CD68 | all | 1.21 (1.03~1.42) | 0.0210* | 0.0347* |
|  |  | Classical | 1.54 (0.95~2.49) | 0.0778 | 0.1864 |
|  |  | Mesenchymal | 1.15 (0.86~1.53) | 0.3520 | 0.7479 |
|  |  | Neural | 1.16 (0.66~2.04) | 0.5950 | 0.2673 |
|  |  | Proneural | 1.05 (0.73~1.53) | 0.7766 | 0.5442 |
| ***Bone marrow monocyte markers*** | SELL | all | 1 (0.89~1.13) | 0.9359 | 0.4683 |
|  |  | Classical | 1.5 (0.87~2.57) | 0.1455 | 0.7612 |
|  |  | Mesenchymal | 1 (0.8~1.25) | 0.9833 | 0.6840 |
|  |  | Neural | 1.64 (0.91~2.98) | 0.1019 | 0.1084 |
|  |  | Proneural | 0.93 (0.76~1.15) | 0.5226 | 0.9084 |
|  | MET | all | 0.93 (0.82~1.06) | 0.2880 | 0.0759 |
|  |  | Classical | 0.97 (0.5~1.91) | 0.9381 | 0.6393 |
|  |  | Mesenchymal | 0.96 (0.76~1.19) | 0.6874 | 0.7844 |
|  |  | Neural | 2.72 (0.79~9.37) | 0.1133 | 0.6147 |
|  |  | Proneural | 0.85 (0.68~1.07) | 0.1788 | 0.2929 |
|  | CCR2 | all | 0.8 (0.55~1.17) | 0.2486 | 0.8291 |
|  |  | Classical | 0.68 (0.11~4.16) | 0.6798 | 0.1327 |
|  |  | Mesenchymal | 0.89 (0.51~1.54) | 0.6736 | 0.4349 |
|  |  | Neural | 0.87 (0.03~21.57) | 0.9310 | 0.9318 |
|  |  | Proneural | 0.51 (0.21~1.25) | 0.1420 | 0.2807 |
|  | CD93 | all | 1.09 (0.92~1.29) | 0.3309 | 0.9132 |
|  |  | Classical | 1.43 (0.83~2.48) | 0.1957 | 0.2839 |
|  |  | Mesenchymal | 1.01 (0.61~1.65) | 0.9841 | 0.8872 |
|  |  | Neural | 1.16 (0.72~1.87) | 0.5347 | 0.4906 |
|  |  | Proneural | 0.9 (0.64~1.26) | 0.5255 | 0.9920 |
|  | KIT | all | 1.02 (0.91~1.14) | 0.7505 | 0.3155 |
|  |  | Classical | 1.2 (0.87~1.67) | 0.2703 | 0.1178 |
|  |  | Mesenchymal | 1.27 (0.86~1.88) | 0.2265 | 0.6425 |
|  |  | Neural | 0.82 (0.51~1.31) | 0.4007 | 0.1416 |
|  |  | Proneural | 1.11 (0.95~1.3) | 0.1994 | 0.0226* |
|  | CLEC12A | all | 0.97 (0.7~1.33) | 0.8351 | 0.6144 |
|  |  | Classical | 9.94 (1.28~77.39) | 0.0282* | 0.8870 |
|  |  | Mesenchymal | 1.24 (0.78~1.97) | 0.3681 | 0.1609 |
|  |  | Neural | 1.23 (0.58~2.6) | 0.5895 | 0.8982 |
|  |  | Proneural | 0.25 (0.06~0.94) | 0.0398* | 0.2082 |
| ***Brainstem microglia markers*** | MERTK | all | 1.12 (0.9~1.39) | 0.3298 | 0.8579 |
|  |  | Classical | 1.34 (0.67~2.68) | 0.4023 | 0.2080 |
|  |  | Mesenchymal | 1.19 (0.74~1.91) | 0.4715 | 0.8624 |
|  |  | Neural | 0.17 (0.03~1.15) | 0.0699 | 0.0088* |
|  |  | Proneural | 0.96 (0.69~1.34) | 0.8234 | 0.5648 |
|  | F11R | all | 1.33 (1.1~1.61) | 0.0036* | 0.0037* |
|  |  | Classical | 0.82 (0.5~1.37) | 0.4555 | 0.7473 |
|  |  | Mesenchymal | 1.05 (0.73~1.49) | 0.8021 | 0.3551 |
|  |  | Neural | 1.64 (0.85~3.15) | 0.1390 | 0.1699 |
|  |  | Proneural | 1.51 (0.96~2.38) | 0.0750 | 0.8571 |
|  | P2RY13 | all | 1.02 (0.9~1.16) | 0.7564 | 0.9129 |
|  |  | Classical | 1.32 (0.88~1.97) | 0.1740 | 0.2778 |
|  |  | Mesenchymal | 1.07 (0.87~1.33) | 0.5153 | 0.6114 |
|  |  | Neural | 0.8 (0.4~1.58) | 0.5163 | 0.3585 |
|  |  | Proneural | 0.96 (0.77~1.2) | 0.7309 | 0.7505 |
|  | CADM1 | all | 1.24 (1.02~1.51) | 0.0281* | 0.0317* |
|  |  | Classical | 0.65 (0.3~1.39) | 0.2681 | 0.1607 |
|  |  | Mesenchymal | 1.09 (0.8~1.47) | 0.5920 | 0.3454 |
|  |  | Neural | 1.03 (0.5~2.15) | 0.9351 | 0.7157 |
|  |  | Proneural | 1.21 (0.84~1.74) | 0.3156 | 0.4809 |
|  | CD81 | all | 1.4 (1~1.97) | 0.0503 | 0.0119* |
|  |  | Classical | 4.34 (1.93~9.74) | 0.0004* | 0.0084* |
|  |  | Mesenchymal | 0.67 (0.38~1.19) | 0.1704 | 0.7523 |
|  |  | Neural | 0.92 (0.38~2.22) | 0.8557 | 0.7215 |
|  |  | Proneural | 1.89 (0.89~4.02) | 0.0994 | 0.8745 |
|  | CX3CR1 | all | 1.08 (0.99~1.19) | 0.0773 | 0.0759 |
|  |  | Classical | 1.15 (0.89~1.49) | 0.2949 | 0.6662 |
|  |  | Mesenchymal | 1.13 (0.96~1.32) | 0.1356 | 0.1188 |
|  |  | Neural | 1.52 (0.83~2.8) | 0.1760 | 0.4361 |
|  |  | Proneural | 1.04 (0.91~1.2) | 0.5527 | 0.4584 |
