# Supplementary material for: F11R Is a Novel Monocyte Prognostic Biomarker for Malignant Glioma
Source: PLoS One. 2013 Oct 11;8(10):e77571. doi: 10.1371/journal.pone.0077571 (PMC3795683; doi:10.1371/journal.pone.0077571)
Supplement: Table S9 — Relative contributions of TCGA subtypes to the survival outcome of GBM patients. Continuous and dichotomized gene expression (high and low expression relative to the median) was analyzed using a multivariate Cox model to account for the relative contributions of specific TCGA subtypes of the GBM samples in the GSE16011 GEO dataset, and hazard ratios (HR), 95% confidence intervals (CI), and associated p-values were generated. (DOC) [file pone.0077571.s014.doc]

***Supplementary Table S9. Relative contributions of TCGA subtypes to the survival outcome of GBM patients. Continuous and dichotomized gene expression (high and low expression relative to the median) was analyzed using a multivariate Cox model to account for the relative contributions of specific TCGA subtypes of the GBM samples in the GSE16011 GEO dataset, and hazard ratios (HR), 95% confidence intervals (CI), and associated p-values were generated.***

| **Category** | **Variable** | **Subtype contribution** | **P-value** | **HR and 95% CI** |
| --- | --- | --- | --- | --- |
| ***General macrophage markers*** | **AIF1: Continuous** |  | 0.0155* | 1.32 (1.05~1.65) |
|  |  | **Subtype (overall)** | 0.0065* |  |
|  |  | **Classical vs. Mesenchymal** | 0.3304 | 1.28 (0.78~2.13) |
|  |  | **Neural vs. Mesenchymal** | 0.4729 | 1.23 (0.7~2.15) |
|  |  | **Proneural vs. Mesenchymal** | 0.0141* | 0.58 (0.37~0.9) |
|  | **AIF1: High vs. Low** |  | 0.0496* | 1.42 (1~2.01) |
|  |  | **Subtype (overall)** | 0.0072* |  |
|  |  | **Classical vs. Mesenchymal** | 0.6153 | 1.13 (0.7~1.81) |
|  |  | **Neural vs. Mesenchymal** | 0.7748 | 1.08 (0.63~1.86) |
|  |  | **Proneural vs. Mesenchymal** | 0.0042* | 0.54 (0.35~0.82) |
|  | **CD68: Continuous** |  | 0.1222 | 1.16 (0.96~1.4) |
|  |  | **Subtype (overall)** | 0.0188* |  |
|  |  | **Classical vs. Mesenchymal** | 0.5798 | 1.15 (0.7~1.9) |
|  |  | **Neural vs. Mesenchymal** | 0.6089 | 1.16 (0.66~2.05) |
|  |  | **Proneural vs. Mesenchymal** | 0.0231* | 0.58 (0.37~0.93) |
|  | **CD68: High vs. Low** |  | 0.0886 | 1.36 (0.95~1.93) |
|  |  | **Subtype (overall)** | 0.0128* |  |
|  |  | **Classical vs. Mesenchymal** | 0.7154 | 1.09 (0.69~1.74) |
|  |  | **Neural vs. Mesenchymal** | 0.6381 | 1.14 (0.65~2) |
|  |  | **Proneural vs. Mesenchymal** | 0.0085* | 0.56 (0.36~0.86) |
| ***Bone marrow monocyte markers*** | **SELL: Continuous** |  | 0.9927 | 1 (0.87~1.15) |
|  |  | **Subtype (overall)** | 0.0063* |  |
|  |  | **Classical vs. Mesenchymal** | 0.8740 | 0.96 (0.59~1.56) |
|  |  | **Neural vs. Mesenchymal** | 0.9819 | 0.99 (0.58~1.7) |
|  |  | **Proneural vs. Mesenchymal** | 0.0014* | 0.5 (0.33~0.76) |
|  | **SELL: High vs. Low** |  | 0.1516 | 1.3 (0.91~1.86) |
|  |  | **Subtype (overall)** | 0.0041* |  |
|  |  | **Classical vs. Mesenchymal** | 0.6766 | 1.11 (0.68~1.8) |
|  |  | **Neural vs. Mesenchymal** | 0.8098 | 1.07 (0.62~1.84) |
|  |  | **Proneural vs. Mesenchymal** | 0.0016* | 0.51 (0.33~0.77) |
|  | **MET: Continuous** |  | 0.2173 | 0.91 (0.79~1.05) |
|  |  | **Subtype (overall)** | 0.0050* |  |
|  |  | **Classical vs. Mesenchymal** | 0.6299 | 0.89 (0.57~1.41) |
|  |  | **Neural vs. Mesenchymal** | 0.7732 | 0.92 (0.54~1.59) |
|  |  | **Proneural vs. Mesenchymal** | 0.0007* | 0.48 (0.31~0.73) |
|  | **MET: High vs. Low** |  | 0.2862 | 1.21 (0.86~1.7) |
|  |  | **Subtype (overall)** | 0.0144* |  |
|  |  | **Classical vs. Mesenchymal** | 0.9156 | 1.03 (0.65~1.62) |
|  |  | **Neural vs. Mesenchymal** | 0.9188 | 1.03 (0.6~1.76) |
|  |  | **Proneural vs. Mesenchymal** | 0.0046* | 0.53 (0.34~0.82) |
|  | **CCR2: Continuous** |  | 0.1457 | 0.73 (0.48~1.11) |
|  |  | **Subtype (overall)** | 0.0040* |  |
|  |  | **Classical vs. Mesenchymal** | 0.5752 | 0.88 (0.56~1.39) |
|  |  | **Neural vs. Mesenchymal** | 0.7317 | 0.91 (0.53~1.57) |
|  |  | **Proneural vs. Mesenchymal** | 0.0005* | 0.47 (0.31~0.72) |
|  | **CCR2: High vs. Low** |  | 0.2451 | 0.82 (0.58~1.15) |
|  |  | **Subtype (overall)** | 0.0036* |  |
|  |  | **Classical vs. Mesenchymal** | 0.7154 | 0.92 (0.59~1.44) |
|  |  | **Neural vs. Mesenchymal** | 0.8481 | 0.95 (0.55~1.63) |
|  |  | **Proneural vs. Mesenchymal** | 0.0006* | 0.47 (0.3~0.72) |
|  | **CD93: Continuous** |  | 0.7902 | 1.03 (0.84~1.26) |
|  |  | **Subtype (overall)** | 0.0088* |  |
|  |  | **Classical vs. Mesenchymal** | 0.9538 | 0.99 (0.61~1.59) |
|  |  | **Neural vs. Mesenchymal** | 0.8987 | 1.04 (0.55~1.97) |
|  |  | **Proneural vs. Mesenchymal** | 0.0044* | 0.51 (0.32~0.81) |
|  | **CD93: High vs. Low** |  | 0.4210 | 0.85 (0.58~1.25) |
|  |  | **Subtype (overall)** | 0.0046* |  |
|  |  | **Classical vs. Mesenchymal** | 0.6546 | 0.9 (0.56~1.44) |
|  |  | **Neural vs. Mesenchymal** | 0.6869 | 0.88 (0.48~1.62) |
|  |  | **Proneural vs. Mesenchymal** | 0.0010* | 0.46 (0.29~0.73) |
|  | **KIT: Continuous** |  | 0.0775 | 1.12 (0.99~1.28) |
|  |  | **Subtype (overall)** | 0.0020* |  |
|  |  | **Classical vs. Mesenchymal** | 0.6767 | 0.91 (0.58~1.42) |
|  |  | **Neural vs. Mesenchymal** | 0.4835 | 0.81 (0.45~1.45) |
|  |  | **Proneural vs. Mesenchymal** | 0.0003* | 0.42 (0.26~0.67) |
|  | **KIT: High vs. Low** |  | 0.0448* | 1.45 (1.01~2.08) |
|  |  | **Subtype (overall)** | 0.0017* |  |
|  |  | **Classical vs. Mesenchymal** | 0.6823 | 0.91 (0.58~1.42) |
|  |  | **Neural vs. Mesenchymal** | 0.4445 | 0.8 (0.45~1.42) |
|  |  | **Proneural vs. Mesenchymal** | 0.0002* | 0.43 (0.28~0.67) |
|  | **CLEC12A: Continuous** |  | 0.7670 | 0.95 (0.68~1.33) |
|  |  | **Subtype (overall)** | 0.0061* |  |
|  |  | **Classical vs. Mesenchymal** | 0.8125 | 0.95 (0.6~1.49) |
|  |  | **Neural vs. Mesenchymal** | 0.9551 | 0.98 (0.58~1.69) |
|  |  | **Proneural vs. Mesenchymal** | 0.0011* | 0.5 (0.33~0.76) |
|  | **CLEC12A: High vs. Low** |  | 0.6674 | 0.93 (0.67~1.3) |
|  |  | **Subtype (overall)** | 0.0061* |  |
|  |  | **Classical vs. Mesenchymal** | 0.8181 | 0.95 (0.61~1.48) |
|  |  | **Neural vs. Mesenchymal** | 0.9088 | 0.97 (0.56~1.67) |
|  |  | **Proneural vs. Mesenchymal** | 0.0010* | 0.5 (0.33~0.75) |
| ***Brainstem microglia markers*** | **MERTK: Continuous** |  | 0.8359 | 1.03 (0.8~1.31) |
|  |  | **Subtype (overall)** | 0.0093* |  |
|  |  | **Classical vs. Mesenchymal** | 0.9174 | 0.98 (0.62~1.55) |
|  |  | **Neural vs. Mesenchymal** | 0.9918 | 1 (0.58~1.7) |
|  |  | **Proneural vs. Mesenchymal** | 0.0022* | 0.51 (0.33~0.78) |
|  | **MERTK: High vs. Low** |  | 0.7936 | 0.95 (0.67~1.35) |
|  |  | **Subtype (overall)** | 0.0062* |  |
|  |  | **Classical vs. Mesenchymal** | 0.8045 | 0.94 (0.59~1.51) |
|  |  | **Neural vs. Mesenchymal** | 0.9886 | 1 (0.58~1.7) |
|  |  | **Proneural vs. Mesenchymal** | 0.0012* | 0.49 (0.32~0.76) |
|  | **F11R: Continuous** |  | 0.2361 | 1.15 (0.91~1.45) |
|  |  | **Subtype (overall)** | 0.0997 |  |
|  |  | **Classical vs. Mesenchymal** | 0.8834 | 0.97 (0.62~1.51) |
|  |  | **Neural vs. Mesenchymal** | 0.7970 | 1.07 (0.62~1.86) |
|  |  | **Proneural vs. Mesenchymal** | 0.0286* | 0.58 (0.36~0.94) |
|  | **F11R: High vs. Low** |  | 0.0189* | 1.57 (1.08~2.29) |
|  |  | **Subtype (overall)** | 0.0787 |  |
|  |  | **Classical vs. Mesenchymal** | 0.5990 | 0.89 (0.57~1.39) |
|  |  | **Neural vs. Mesenchymal** | 0.6608 | 1.13 (0.66~1.95) |
|  |  | **Proneural vs. Mesenchymal** | 0.0239* | 0.6 (0.38~0.93) |
|  | **P2RY13: Continuous** |  | 0.7333 | 1.02 (0.89~1.18) |
|  |  | **Subtype (overall)** | 0.0063* |  |
|  |  | **Classical vs. Mesenchymal** | 0.9346 | 0.98 (0.62~1.55) |
|  |  | **Neural vs. Mesenchymal** | 0.9759 | 0.99 (0.58~1.69) |
|  |  | **Proneural vs. Mesenchymal** | 0.0013* | 0.5 (0.33~0.76) |
|  | **P2RY13: High vs. Low** |  | 0.7710 | 1.05 (0.75~1.46) |
|  |  | **Subtype (overall)** | 0.0062* |  |
|  |  | **Classical vs. Mesenchymal** | 0.8982 | 0.97 (0.62~1.52) |
|  |  | **Neural vs. Mesenchymal** | 0.9569 | 0.99 (0.58~1.69) |
|  |  | **Proneural vs. Mesenchymal** | 0.0011* | 0.5 (0.33~0.76) |
|  | **CADM1: Continuous** |  | 0.3390 | 1.11 (0.9~1.36) |
|  |  | **Subtype (overall)** | 0.0313* |  |
|  |  | **Classical vs. Mesenchymal** | 0.7706 | 0.94 (0.6~1.46) |
|  |  | **Neural vs. Mesenchymal** | 0.9178 | 0.97 (0.57~1.66) |
|  |  | **Proneural vs. Mesenchymal** | 0.0043* | 0.53 (0.34~0.82) |
|  | **CADM1: High vs. Low** |  | 0.2701 | 1.22 (0.86~1.73) |
|  |  | **Subtype (overall)** | 0.0233* |  |
|  |  | **Classical vs. Mesenchymal** | 0.6675 | 0.91 (0.57~1.43) |
|  |  | **Neural vs. Mesenchymal** | 0.8274 | 0.94 (0.55~1.62) |
|  |  | **Proneural vs. Mesenchymal** | 0.0027* | 0.52 (0.34~0.8) |
|  | **CD81: Continuous** |  | 0.1766 | 1.27 (0.9~1.8) |
|  |  | **Subtype (overall)** | 0.0146* |  |
|  |  | **Classical vs. Mesenchymal** | 0.8222 | 1.05 (0.66~1.67) |
|  |  | **Neural vs. Mesenchymal** | 0.8793 | 1.04 (0.61~1.79) |
|  |  | **Proneural vs. Mesenchymal** | 0.0057* | 0.54 (0.35~0.84) |
|  | **CD81: High vs. Low** |  | 0.1165 | 1.32 (0.93~1.86) |
|  |  | **Subtype (overall)** | 0.0254* |  |
|  |  | **Classical vs. Mesenchymal** | 0.8830 | 1.03 (0.66~1.63) |
|  |  | **Neural vs. Mesenchymal** | 0.9870 | 1 (0.58~1.7) |
|  |  | **Proneural vs. Mesenchymal** | 0.0070* | 0.55 (0.36~0.85) |
|  | **CX3CR1: Continuous** |  | 0.0758 | 1.09 (0.99~1.2) |
|  |  | **Subtype (overall)** | 0.0062* |  |
|  |  | **Classical vs. Mesenchymal** | 0.9950 | 1 (0.64~1.56) |
|  |  | **Neural vs. Mesenchymal** | 0.9265 | 0.98 (0.57~1.66) |
|  |  | **Proneural vs. Mesenchymal** | 0.0013* | 0.5 (0.33~0.76) |
|  | **CX3CR1: High vs. Low** |  | 0.0804 | 1.34 (0.97~1.86) |
|  |  | **Subtype (overall)** | 0.0055* |  |
|  |  | **Classical vs. Mesenchymal** | 0.9266 | 0.98 (0.63~1.52) |
|  |  | **Neural vs. Mesenchymal** | 0.9530 | 0.98 (0.58~1.68) |
|  |  | **Proneural vs. Mesenchymal** | 0.0010* | 0.49 (0.32~0.75) |
